# Applying the auxin-based degron system for the inducible, reversible and complete protein degradation in *Komagataella phaffii*

**DOI:** 10.1016/j.isci.2022.104888

**Published:** 2022-08-06

**Authors:** Leonie Lehmayer, Lukas Bernauer, Anita Emmerstorfer-Augustin

**Affiliations:** 1 Institute of Molecular Biotechnology, Graz University of Technology, NAWI Graz, Petersgasse 14/II, 8010 Graz, Austria; 2 BioTechMed-Graz, 8010 Graz, Austria; 3 acib - Austrian Centre of Industrial Biotechnology, 8010 Graz, Austria

**Keywords:** molecular biology, microbiology

## Abstract

The auxin-inducible degron (AID) system is a useful technique to rapidly deplete any protein of interest “on-demand.” In this study, we successfully established the AID system for the “biotech” yeast *Komagataella phaffii*. First, we tested different expression levels of TIR1 for auxin-induced degradation of the glycerol kinase Gut1. Moderate expression of TIR1 resulted in complete degradation of the target protein within several minutes. Second, we show that the absence of all three Wsc type sensors is detrimental to cell growth, which indicates that these are the dominant cell wall sensors this yeast. Third, down-regulation of Erg1, an essential enzyme of the ergosterol biosynthetic pathway, resulted in quick and efficient accumulation of squalene, a pharmaceutically relevant reagent. We conclude that AID is an extremely powerful tool that, for the first time, enables the analysis of gene essentiality and function in *K. phaffii*.

## Highlights

• Conditional AID mutants are generated in *Komagataella phaffii* expressing OsTIR1
• Target proteins fused to AID are depleted rapidly on the addition of auxin
• The deletion of all three Wsc-type severely reduces the growth of *K. phaffii*
• Cells degrading Erg1 quickly and efficiently accumulated squalene

## Graphical abstract

**Figure:**
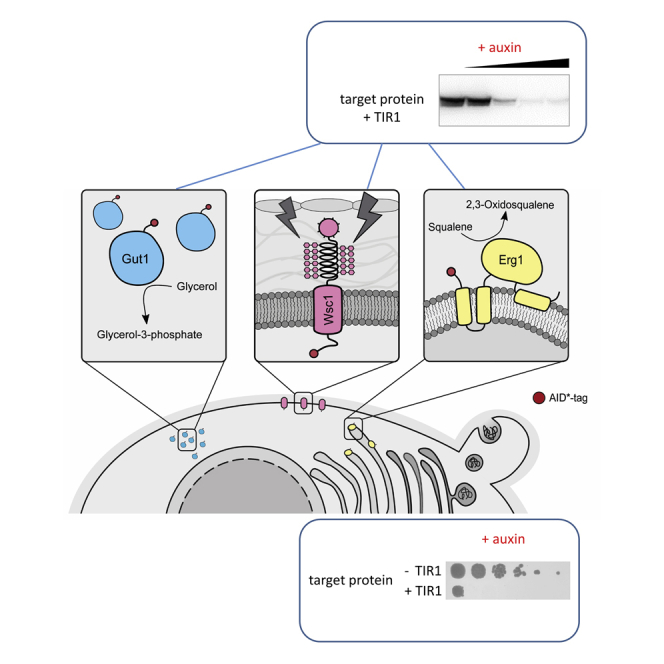

## Introduction

*Komagataella phaffii* (syn. *Pichia pastoris*) is a methylotrophic budding yeast that is known best for its extensive use as efficient protein production and secretion host in industrial applications. Lately, it started to gain attention as attractive alternative model yeast in fundamental research (Bernauer et al., 2021). Although the genetic engineering of this yeast was substantially facilitated by novel tools such as CRISPR/Cas9 (Weninger et al., 2018) and the construction of stable heterothallic strains (Heistinger et al., 2017), its use in fundamental research is still underrepresented owing to a critical lack of advanced technologies. In contrast, many highly useful methods have been developed for *S. cerevisiae* like tetrad analysis, yeast one and two hybrid screening assays, or CRISPRi, diverse sets of conditional mutants, and gene knockout libraries, most of which have not been implemented for other yeasts yet.

One effective strategy for the analysis of protein function and the biological process influenced by that protein is the simple generation of a gene knockout. However, a gene knockout strategy cannot be applied to genes essential for growth. Conditional mutants of a gene, as for example temperature-sensitive (ts) mutants, are effective tools in the analysis of essential genes, but such mutants have to be screened for and frequently show hypomorphic phenotypes of the gene (Hartwell, 1967; Tan et al., 2009). Additionally, the investigation of ts mutants requires cells to be propagated at elevated temperatures, which not only causes inactivation of the respective protein but also activates diverse cellular stress response pathways (Trott and Morano, 2007). Quite recently, a highly powerful tool for the proteolytic elimination of a target protein, the auxin-inducible degron (AID) method has been developed (Nishimura et al., 2009). This system uses the plant hormone auxin, its *in vivo* binding target, IAA17 (the AID-tag), and its adaptor for E3 ubiquitin ligase, TIR1. To generate AID-based conditional knockout strains, a target gene fused to the AID-tag sequence must be integrated into the endogenous target gene locus by homologous recombination. Ectopically expressed TIR1 protein allows cells to rapidly degrade AID-fused target proteins on the addition of auxin. This system has the huge advantage that it can be applied universally to any eukaryotic cell, given the prerequisite that TIR1 can be expressed functionally and that fusion of the AID-tag to the protein of interest does not disturb its function.

In order to establish the AID-system in *K. phaffii*, we constructed robust integration plasmids expressing TIR1 from promoters of different strengths that can easily be transformed and efficiently be integrated into any *K. phaffii* strain expressing a target gene fused to an AID-tag. Using a shorter and better stable version of the AID-tag, AID∗ (Morawska and Ulrich, 2013), we tagged Gut1, a glycerol kinase important for the growth of glycerol. This kinase has often been targeted, e.g. for testing Cre/lox knockout strategies (Näätsaari et al., 2012) and CRISPR/Cas9 systems in *K. phaffii* (Näätsaari et al., 2012), because of its clear growth phenotype on glycerol media.

In order to test the AID technology in a scientific question, we focused on transmembrane cell wall integrity (CWI) sensors. CWI sensors can detect stress as a mechanical change in the cell wall, which causes conformational changes in the sensors, activation of a complex MAP kinase pathway and, ultimately, remodeling of the cell wall (reviewed by (Jendretzki et al., 2011)). In *Saccharomyces cerevisiae*, five transmembrane sensors have been characterized and allocated to two families, the Wsc-type and the Mid-type family (reviewed by (Kock et al., 2015)). The Wsc-type family includes three cell wall sensors named Wsc1 (protein ID NP_014650.1), Wsc2 (protein ID NP_014116.1), and Wsc3 (protein ID NP_014536.1), where Wsc2 and Wsc3 are paralogous. The Mid-type family includes the two paralogous sensors Mid2 (protein ID NP_013436.1) and Mtl1 (protein ID NP_011537.1). Interestingly, *K. phaffii* is the only yeast investigated so far, where only homologous proteins for the three Wsc-type sensors Wsc1 (protein ID CCA39485.1), Wsc2 (protein ID CCA37335.1), and its paralogue Wsc3 (protein ID CCA37334.1) (Ohsawa et al., 2017), but no homologous proteins of Mid-type CWI sensors could be found according to a BLAST search. This poses the question, of whether Wsc-type sensors suffice for monitoring cell wall integrity in *K. phaffii*. Therefore, we used a *wsc2*Δ *wsc3*Δ double knockout strain for auxin-induced degradation of Wsc1-AID∗-3HA and monitored cell growth. As the CWI pathway and cell wall sensors are essential in all fungi, the absence of all three Wsc sensors is expected to severely impact cell growth.

And last, we aimed to implement AID-based protein down-regulation in an industrial application. A currently hotly debated topic is the rising demand for squalene, a polyunsaturated triterpenoid that acts as a precursor for the biosynthesis of all sterols in yeasts, animals, and plants (Spanova and Daum, 2011). Owing to its unique structural characteristics, squalene is an important component of many skincare products, but also of parenteral emulsions for drug and vaccine delivery (Fox, 2009). A larger proportion of this substance is extracted from the liver of sharks (Hernández-Pérez et al., 1997), which poses a major risk to shark populations and the protection of marine wildlife (Macdonald and Soll, 2020). As intermediate product of the yeast ergosterol biosynthesis pathway, squalene can be accumulated in yeast by down-regulation of squalene monooxygenase Erg1 (Garaiová et al., 2014). As Erg1 is an essential protein, this step has to be modulated carefully in order to balance cellular growth and squalene production. Therefore, we have chosen Erg1 as a target protein for auxin-induced degradation to monitor squalene levels over time.

In this work, we report the first use of the AID system in *K. phaffii*. We show that diverse cellular processes can be targeted: glycerol metabolism, stress signaling pathways like the cell-wall integrity pathway and essential biosynthetic genes like *ERG1*. All targets were chosen carefully in order to cover a broad range of aspects, like protein localization, abundance, and activity. In all cases, the target protein retained its function upon AID∗-tagging and, depending on its expression level and localization was degraded with half-lives of 10–40 min.

## Results

### High expression levels of TIR1 cause basal degradation of Gut1-AID∗-3HA

**Figure 1:**
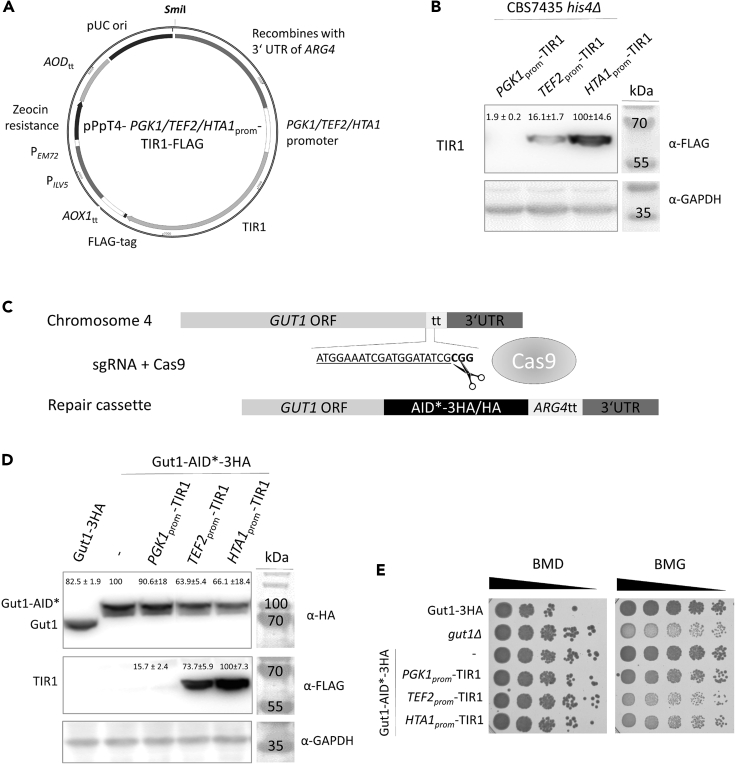
TIR1 expression levels influence protein stability of Gut1-AID∗-3HA (A) Vectors expressing TIR1 are linearized with *Smi*I to integrate into the 5‘UTR of the *ARG4* locus of *K. phaffii*. (B) Strain CBS7435 *his4*Δ expressing TIR1 either from *PGK1*_prom_ (yLL132), *TEF2*_prom_ (yLL108), or *HTA1*_prom_ (yLL109) were grown to middle exponential phase at 28°C, harvested, lysed, and proteins were extracted, resolved by SDS–PAGE, and analyzed by immunoblotting with anti-FLAG antibody, as described under STAR Methods. Loading control, GAPDH detected on the same immunoblots using anti-GAPDH antibody. MW, marker proteins (kDa). Values above the lanes represent the percentage of relative TIR1 levels (average of three independent experiments with SEM). (C) A CRISPR/Cas genome engineering approach was used for C-terminal AID∗- and HA-tagging of *GUT1*. The targeted guide RNA binding site and PAM sequence are located downstream of the *GUT1* ORF. The double-strand break caused by Cas9 activity is repaired by a repair cassette harboring up- and downstream sequences homologous to *GUT1*, an AID∗-3HA or 3HA tag, the transcription termination sequence of *ARG4* and the 5’UTR of *GUT1*. (D) A CBS7435 *his4*Δ strain expressing either *GUT1*-3HA (yAEA398), GUT1-AID∗-3HA (yLL116), or otherwise isogenic derivatives co-expressing TIR1 either from *PGK1*_prom_ (yLL118), *TEF2*_prom_ (yLL120), or *HTA1*_prom_ (yLL122) were grown to middle exponential phase at 28 °C, harvested, lysed, and proteins were extracted, resolved by SDS–PAGE, and analyzed by immunoblotting with anti-HA and anti-FLAG antibody, as described under STAR Methods. Loading control, GAPDH detected on the same immunoblots using anti-GAPDH antibody. MW, marker proteins (kDa). Values above the lanes represent the percentage of relative Gut1-3HA and Gut1-AID∗-3HA levels with and without co-expression of TIR1 (average of three independent experiments with SEM). (E) Strain yP322 (CBS7435 *gut1*Δ) and the same strains as in D were cultivated as described in STAR Methods, and then samples of a set of 5-fold serial dilutions were spotted using a multiprong inoculator on an agar plate containing either BMD or BMG, and, after incubation for 72 h at 28°C, the resulting growth was recorded.

In order to examine whether TIR1 can be expressed in *K. phaffii*, we constructed simple and universally applicable integration plasmids based on the pPpT4 plasmid family (Näätsaari et al., 2012). A TIR1 originating from *Oryza sativa* and codon-optimized for expression in *S. cerevisiae* was used (NBRP ID: BYP7569; Masato Kanemaki) for cloning and a FLAG-tag was added C-terminally for immunoblot detection. In order to reach different expression levels of TIR1, we used the *K. phaffii* endogenous *PGK1* (protein ID CCA37205.1), *TEF2* (homolog of *S. cerevisiae TEF1*) (protein ID CCA37646.1), and *HTA1* (Protein ID SCV12077.1) promoters (Figure 1A). *PGK1*_prom_ and *TEF2*_prom_ are well-known, rather moderate promoters often used for low-to intermediate-level expression in *S. cerevisiae* (Partow et al., 2010). *HTA1*_prom_ is the promoter of histone H2A and, therefore, is expected to be a very strong and stably expressed promoter that is constitutively active and less dependent on any growth phase or a specific carbon source (Vogl et al., 2018). Promoter strengths influenced expression levels of TIR1 as expected: *PGK1*_prom_ resulted in very low expression of TIR1, whereas *TEF2*_prom_ and *HTA1*_prom_ clearly upregulated expression of TIR1 (Figure 1B). Using a combination of AID tagging with CRISPR/Cas (Figure 1C), we generated conditional alleles of *GUT1.* In order to spatially separate the AID∗-tag from the C-terminus of the tagged target protein, we added a 12 amino acid long flexible GS linker to all our constructs (Table S1) (all plasmid maps can be downloaded as supplemental information). Other studies have shown that CRISPR-mediated AID-tagging of target proteins can be problematic in mammalian cells already expressing TIR1, most probably owing to reduced cellular fitness caused by immediate basal degradation of the target protein (Natsume et al., 2016). Hence, we first checked the influence of different TIR1 expression levels on basal degradation of Gut1-AID∗-3HA. Overexpression of TIR1 reduced protein stability of Gut1-AID∗-3HA by 30–40% even without the addition of auxin as shown by immunoblot detection (Figure 1D) and caused growth defects similar to a *gut1*Δ strain on glycerol media (Figure 1E). AID-tagging of Gut1 did not disturb its function as confirmed by wild-type-like growth on glycerol when present as the sole carbon source.

### Gut1-AID∗-3HA is rapidly depleted upon the addition of auxin

**Figure 2:**
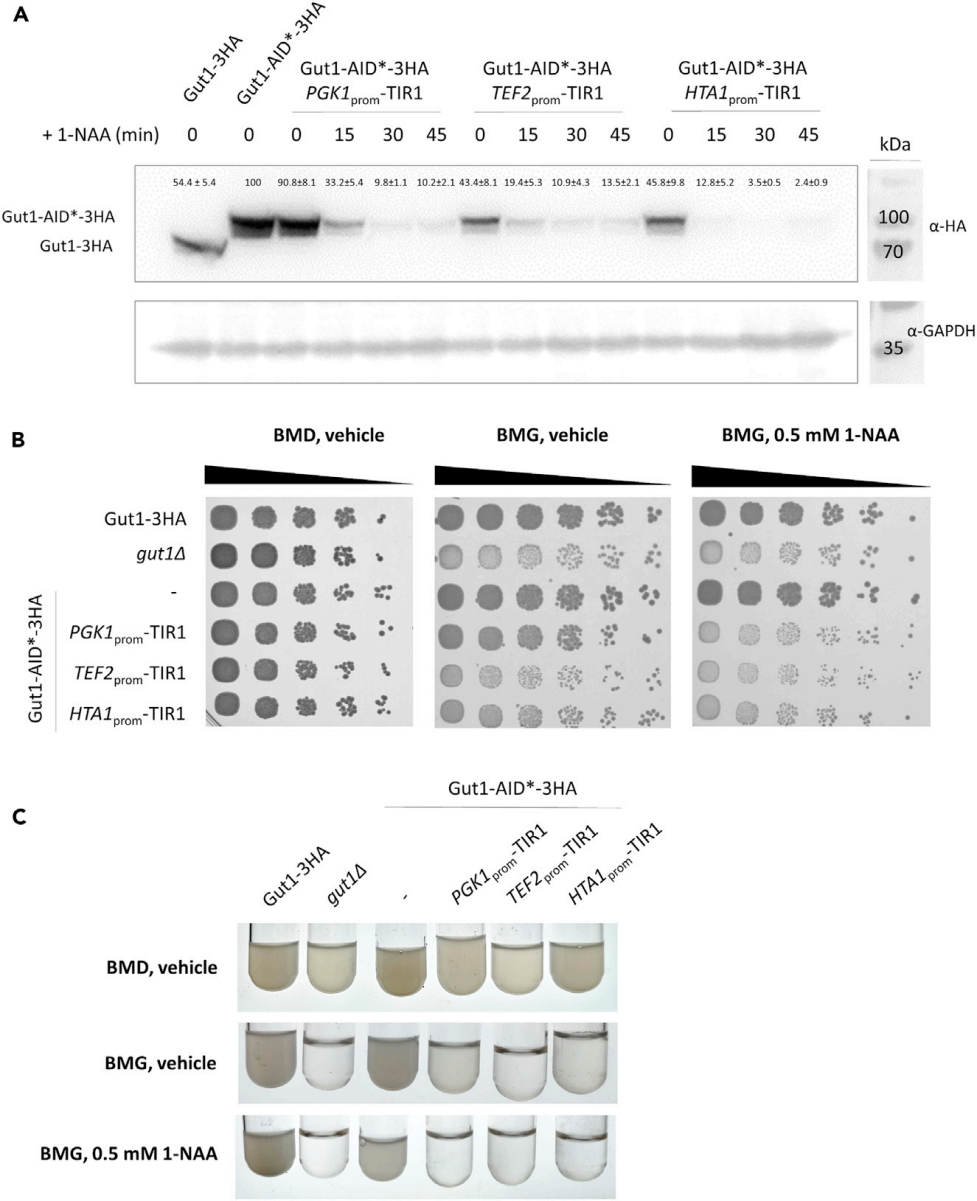
Auxin-induction causes rapid depletion of Gut1-AID∗-3HA (A) A CBS7435 *his4*Δ strain expressing either Gut1-3HA (yAEA398), Gut1-AID∗-3HA (yLL116), or otherwise isogenic derivatives co-expressing TIR1 from *PGK1*_prom_ (yLL118), *TEF2*_prom_ (yLL120), or *HTA1*_prom_ (yLL122) were grown to middle exponential phase at 28°C, harvested, lysed, and proteins were extracted, resolved by SDS–PAGE, and analyzed by immunoblotting with anti-HA, as described under STAR Methods. Loading control, GAPDH detected on the same immunoblots using anti-GAPDH antibody. MW, marker proteins (kDa). Signal quantifications are presented as mean +/–SEM. (B) Strain yP322 (CBS7435 *gut1*Δ) and the same strains as in A were cultivated as described in STAR Methods, and then samples of a set of 5-fold serial dilutions were spotted using a multiprong inoculator on agar plates containing either BMG with vehicle alone (DMSO), or 0.5 mM 1-NAA in DMSO, and, after incubation for 72 h at 28 °C, the resulting growth was recorded. (C) The same strains as in B were cultivated in 2 mL of BMD with vehicle alone (DMSO), BMG with vehicle alone (DMSO) or BMG with 0.5 mM 1-NAA in DMSO overnight, and the resulting growth was recorded.

Next, we demonstrated that Gut1-AID∗-3HA strains expressing TIR1 from all three promoters rapidly and efficiently degraded the target protein in an auxin-induced manner (Figure 2A). The depletion of degron-tagged proteins can be achieved using either the natural form of auxin, IAA (indole-3-acetic acid), or a synthetic version, 1-NAA (naphthalene-acetic acid), whereas both IAA and 1-NAA can be reliably used to deplete proteins in yeast (Gopalakrishnan et al., 2019). It was shown that different yeast strains have different sensitivities to auxin (Shetty et al., 2019), which is why we empirically determined the most suitable concentration of 1-NAA and IAA and found 100 μM of either auxin to suffice for robust and quick degradation of Gut1-AID∗-3HA. Overall, IAA worked better than NAA, especially at low concentrations (Figure S1). However, cell growth was affected by higher concentrations of IAA, whereas 1-NAA did not have the same effect. As efficient degradation of other target proteins investigated in this study benefited from higher concentrations of 1-NAA (Figure S1), we used 0.5 – 1mM 1-NAA in all further experiments. Auxin-induced depletion of Gut1-AID∗-3HA exhibited good dynamics (approx. 30–45 min) (Figure 2A), well below the doubling time of yeast cells growing on glucose (approx. 100 min). As expected, cells harboring higher levels of TIR1 degraded Gut1-AID∗-3HA faster. Similar to a *gut1*Δ control strain, auxin-induced degradation of Gut1-AID∗-3HA caused a reduced growth phenotype on glycerol as shown on BMG-plates (Figure 2B) and in liquid BMG-media (Figure 2C), whereas the effect was more severe in liquid media. These results are in accordance with data published by the Glieder lab where the deletion of *gut1*Δ caused severe growth defects in liquid media (Näätsaari et al., 2012), and reduced growth on plates containing glycerol as a sole carbon source (Weninger et al., 2016).

### Investigation of cell wall sensors Wsc1, Wsc2, and Wsc3

**Figure 3:**
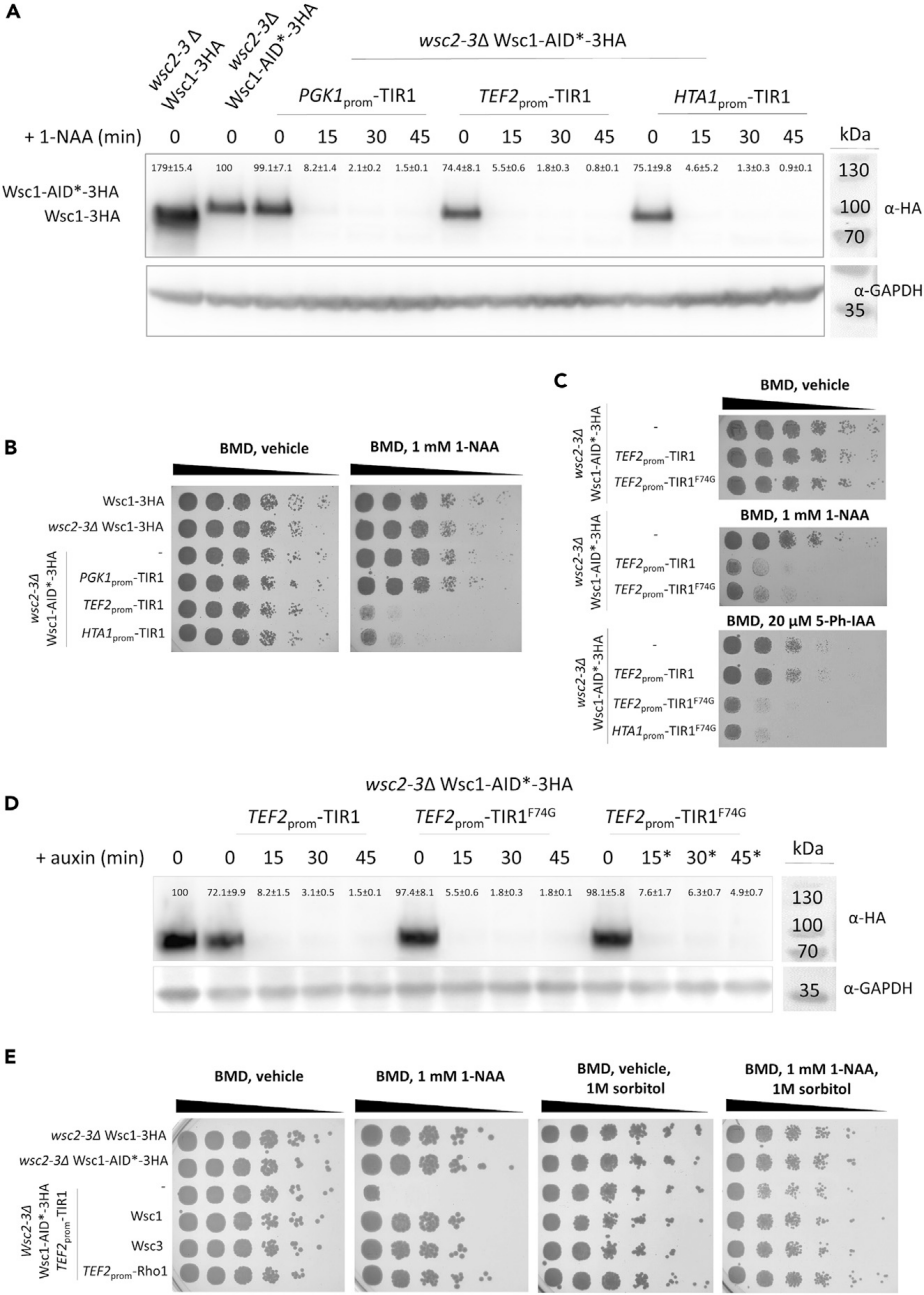
Degradation of Wsc1 in a *wsc2*Δ *wsc3*Δ strain leads to severe growth defects (A) A CBS7435 *wsc2*Δ *wsc3*Δ (*wsc2-3*Δ) knockout strain expressing either Wsc1-3HA (yLL146), Wsc1-AID∗-3HA (yAEA400), or otherwise isogenic derivatives co-expressing TIR1 from *PGK1*_prom_ (yLL141), *TEF2*_prom_ (yLL142), or *HTA1*_prom_ (yLL143) were grown to middle exponential phase at 28°C, harvested, and lysed, and proteins were analyzed by immunoblotting with anti-HA antibody, as described under STAR Methods. Loading control, GAPDH detected on the same immunoblots using anti-GAPDH antibody. MW, marker proteins (kDa). Signal quantifications are presented as mean +/–SEM. (B) The same strains as in A were cultivated as described in STAR Methods, and then samples of a set of 5-fold serial dilutions were spotted using a multiprong inoculator on agar plates containing either BMG with vehicle alone (DMSO) or 1 mM 1-NAA in DMSO, and, after incubation for 72 h at 28°C, the resulting growth was recorded. (C) In order to test AID2, strains Wsc1-AID∗-3HA (yAEA400) or otherwise isogenic derivatives co-expressing TIR1 (yLL142), or TIR1^F74G^ either from *TEF2*_prom_ (yLB215) or *HTA1*_prom_ (yLB218) were cultivated, diluted and spotted on agar plates containing vehicle (DMSO), 1 mM 1-NAA in DMSO, or 20 μM 5-Ph-IAA as described under C. (D) To assess proper the degradation of Wsc1-AID∗-3HA, the same strains as in C were cultivated, protein extracts generated and analyzed by immunoblotting with anti-HA antibody, as described in STAR Methods. Loading control, GAPDH detected on the same immunoblots using anti-GAPDH antibody. MW, marker proteins (kDa). ∗5 μM 5-Ph-IAA used for degradation instead of 0.5 mM 1-NAA. Signal quantifications are presented as mean +/–SEM. (E) A CBS7435 control strain, CBS7435 *WSC1*-3HA (yLL110), *wsc2*Δ *wsc3*Δ (*wsc2-3*Δ) *WSC1*-AID∗-3HA *PGK1*_prom_-TIR1 (yLL141), and otherwise isogenic derivatives thereof co-expressing Wsc1 (yLL151), or Wsc3 (yLL120) from their native promoters, or Rho1 from the *TEF2* promoter (yLL145) as integrated cassettes from the *his*4Δ locus were grown, spotted and incubated on agar plates containing either BMG with vehicle alone (DMSO), 1 mM 1-NAA in DMSO, or 1 M sorbitol w/o vehicle and 1 mM 1-NAA as explained under B.

In order to investigate Wsc-type sensors in *K. phaffii*, we used a *wsc2*Δ *wsc3*Δ double deletion strain to AID∗-tag *WSC1*, and examined, whether auxin-induced degradation of Wsc1-AID∗-3HA leads to a growth phenotype of the strain. As *WSC3* and *WSC2* are located right next to each other on the *K. phaffii* genome, the double-knockout can be created by using only one knockout cassette and was named *wsc2-3*Δ. In all TIR1-expressing *wsc2-3*Δ strains, auxin-induced degradation of Wsc1-AID∗-3HA was highly efficient. After 15 min, barely any signal could be detected in an immunoblot (Figure 3A). Again, strong expression of TIR1 from *TEF2* and *HTA1* promoters triggered basal degradation of Wsc1-AID∗-3HA (Figure S2A), but not enough to cause a growth phenotype on BMD plates (Figure 3B). When *wsc2*Δ *wsc3*Δ Wsc1-AID∗-3HA strains were spotted on auxin plates, a clear growth defect could be observed in strains with moderate to high expression of TIR1 (Figure 3B), and the growth defect was more pronounced on plates containing higher auxin concentrations (Figure S1). In order to test, whether the degradation of Wsc1 was not sufficient, we additionally tested auxin-inducible degron 2 (AID2) (Nishimura et al., 2020; Yesbolatova et al., 2020). AID2 employs an OsTIR1^F74G^ mutant and an auxin derivative, 5-Ph-IAA, and it has the advantage that it shows no detectable leaky degradation, requires a 1000-times lower ligand concentration and was shown to achieve even quicker degradation than the conventional AID. In our case, AID2 reduced the growth of the *wsc2*Δ *wsc3*Δ Wsc1-AID∗-3HA strain to a similar extent to OsTIR1 (Figure 3C). Overall, basal degradation of Wsc1-AID∗-3HA was fully eliminated when co-expressed with OsTIR1^F74G^ instead of OsTIR1, and Wsc1-AID∗-3HA was degraded quickly and efficiently (Figure 3D).

To additionally challenge, whether or not a *wsc1*Δ *wsc2*Δ *wsc3*Δ triple deletion strain is viable, a complementation experiment was designed. Therefore, a pPpZeo-Cas9 plasmid targeting *WSC3* and co-expressing *WSC1* (pPpZeo-Cas9-*wsc3*Δ_*WSC1*, Figure S3A) were used for the transformation of a *wsc1*Δ *wsc2*Δ double delete strain. In *K. phaffii*, non-homologous end-joining is the preferred DNA repair mechanism, which is the reason why frameshift mutations occur after a CRISPR/Cas9-induced double-strand break (Weninger et al., 2016, 2018). Initially, pPpZeo-Cas9 and pPpHyg-Cas9-plasmids were designed to show very low stability, because it is very important for cells to lose Cas9 activity after cell engineering to avoid unspecific mutations in the genome. As a consequence, after transformation and Cas9-induced frameshift mutation of *WSC3* (*wsc3-1*), control cells rapidly lost the plasmid when propagated without selection pressure (Figure S3C). However, in *wsc1*Δ *wsc2*Δ *wsc3-1* cells, the pPpZeo-Cas9-*wsc3*Δ_*WSC1* was retained, even though no selection pressure was applied. As a control, we also used a Cas9 plasmid targeting *GUT1*, and this plasmid could easily be lost by the wild-type and *wsc1*Δ *wsc2*Δ strain.

In *S. pombe*, a double deletion strain of its main functional cell wall sensors, *Sp*Wsc1 and *Sp*Mtl2, was not viable and this defect could be rescued upon mild overexpression of *Sp*Rho1, a downstream GTPase of the cell wall integrity pathway (Cruz et al., 2013). To test, whether the loss of all Wsc proteins could be rescued, we also tested the expression of Wsc1 and Wsc3, and overexpression of an extra allele of *K. phaffii* Rho1 (protein ID CCA40030.2) from the *his4*Δ locus, respectively, which fully complemented the observed growth defects caused by Wsc1 degradation in a *wsc2*Δ *wsc3*Δ double delete (Figure 3E). As a control, we also spotted cells on plates containing 1 M sorbitol as an osmotic stabilizer, which was shown to compensate the growth defects caused by cell wall integrity mutants in *S. cerevisiae* (Verna and Ballester, 1999), and we observed the same effect (Figure 3E). Altogether, our results prove that the loss of all three Wsc-sensors, either degron-induced or by applying a CRISPR/Cas9-based gene deletion and complementation approach leads to severe growth defects that need to be rescued to allow for proper cellular growth.

### Auxin-induced degradation of Erg1 causes accumulation of squalene in the cell

**Figure 4:**
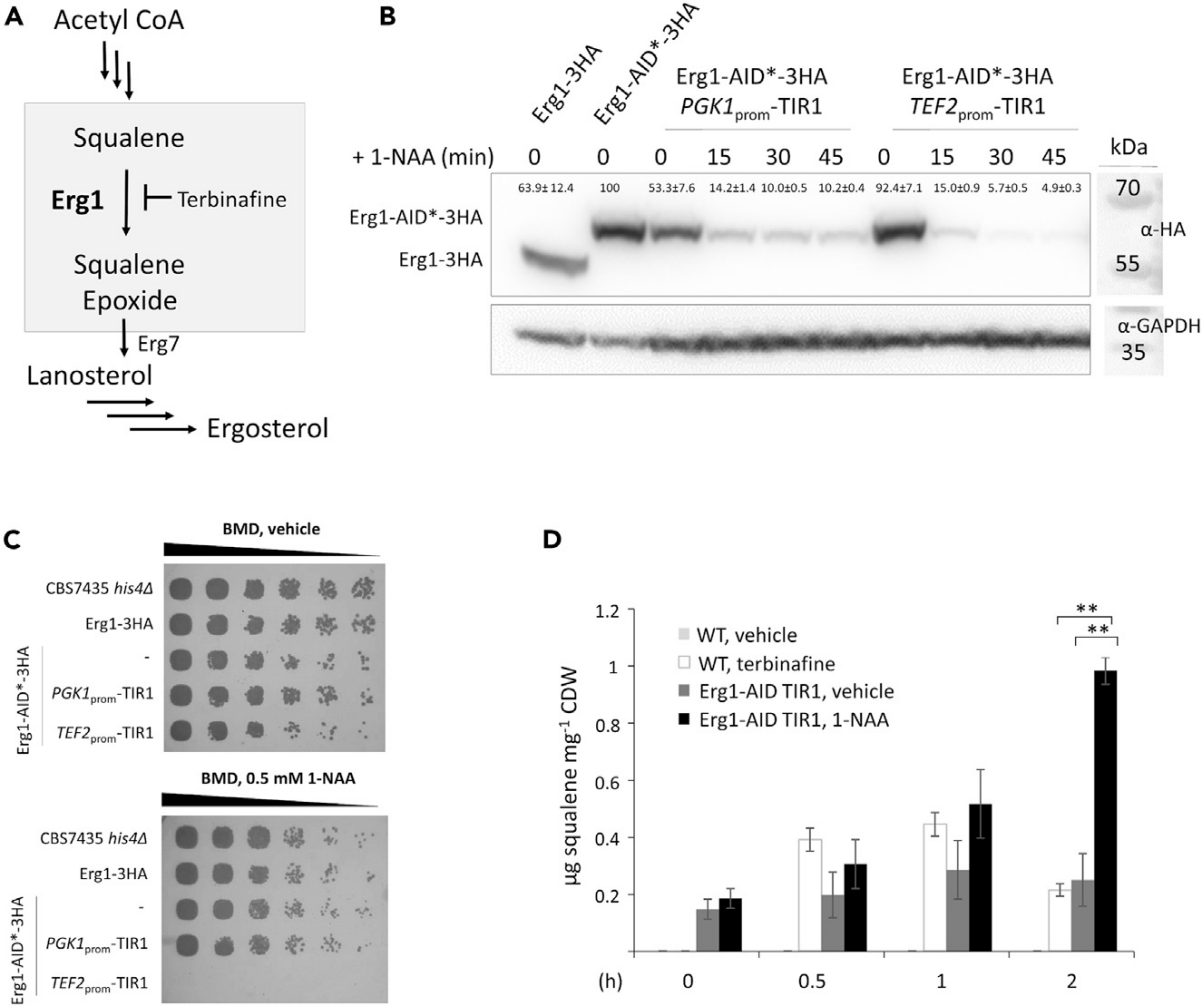
Degradation of Erg1 causes lethality and an accumulation of squalene in the cell (A) Scheme showing details of the ergosterol biosynthesis pathway. The gray box highlights the conversion of squalene to squalene epoxide which is catalyzed by squalene monooxygenase Erg1 and can be inhibited by terbinafine. (B) CBS7435 strains expressing Erg1-3HA (yLL144), Erg1-AID∗-3HA (yLL150), Erg1-AID∗-3HA *PGK1*_prom_-TIR1 (yLL147) and Erg1-AID∗-3HA *TEF2*_prom_-TIR1 (yLL148) were grown to A_600_ ∼ 4 at 28°C, prepared and analyzed by immunoblotting with anti-HA, as described under STAR Methods. GAPDH detected on the same immunoblots using anti-GAPDH antibody was used as a loading control. MW, marker proteins (kDa). Signal quantifications are presented as mean +/–SEM. (C) A CBS7435 control strain and the same strains as in A were grown overnight to saturation, adjusted to A_600_ = 1, and then samples of a set of 5-fold serial dilutions were spotted on agar plates containing either BMG with vehicle alone (DMSO) or 0.5 mM 1-NAA in DMSO. (D) A CBS7435 wild-type strain and a CBS7435 strain expressing Erg1-AID∗-3HA *TEF2*_prom_-TIR1 (yLL148) were grown to A_600_ ∼ 4 at 28 °C, treated with vehicle (DMSO), 0.1 μg mL^−1^ terbinafine or 0.5 mM 1-NAA for the times indicated and squalene levels determined as described in STAR Methods (three technical replicates were performed for each). Quantification of squalene was performed by comparing values to standard calibration curves, and all data were normalized by the internal standard cholesterol and cell dry weight (CDW). Squalene amounts ranging from 0 to 1.2 μg mg^−1^ CDW are represented on the y axis. Error bars, SEM, ∗∗p value < 0.0001, determined by two-tailed Student’s *t* test. *p* values were obtained for samples Erg1-AID treated with 1-NAA compared to the WT treated with terbinafine, and Erg1-AID treated with vehicle, at time point 2 h, respectively).

As mentioned before, *ERG1* is an essential gene of the ergosterol biosynthesis pathway, and the protein is efficiently inhibited by terbinafine (Ryder, 1992) (Figure 4A). Again, a strain producing Erg1-AID∗-3HA from its endogenous locus was transformed with cassettes expressing TIR1 either from the *PGK1*, *TEF2*, or *HTA1* promoters. Even after several rounds of transformation, we were unable to obtain viable Erg1-AID∗-3HA mutants expressing TIR1 from the strong *HTA1* promoter, which indicates that high levels of basal degradation of Erg1-AID∗-3HA may lead to cell death. Proceeding with auxin-induced degradation of Erg1-AID∗-3HA in the other two strains, we noticed that low expression of TIR1 from the *PGK1* promoter rapidly reduced Erg1-AID∗-3HA levels, but only to a certain extent (Figure 4B), which was still sufficient for proper cell growth in spot assays (Figure 4C). Higher expression levels of TIR1 from the *TEF2* promoter led to complete auxin-induced degradation of Erg1-AID∗-3HA within 15–30 min (Figure 4B), which ultimately resulted in cell death (Figure 4C). Basal degradation only happened to a minor extent, reduced overall protein levels by ∼13% (Figure S2B), and did not lead to any growth phenotype (Figure S1C).

Moser et al. published that, in contrast to *S. cerevisiae*, there is barely any squalene found in wild-type *K. phaffii* cells, presumably owing to the quick conversion of this intermediate by Erg1 (Moser et al., 2018, 2020). Although AID∗-tagging of Erg1 slightly increased protein levels compared to Erg1-3HA by ∼45% (Figure S4), it also led to an increase of squalene even in the absence of auxin (Figure 4D), which indicates that C-terminal AID∗-tagging of Erg1 may influence its activity, function or localization. As expected, terbinafine efficiently inhibited Erg1 within 30 min of incubation as monitored by an increase in squalene levels. However, auxin-induced degradation of Erg1-AID∗-3HA worked even more efficiently with squalene levels increased ∼2.5-times over the terbinafine control after 2 h of incubation (Figure 4D).

### Auxin-inducible degron-mediated degradation of Erg1 is rapidly reversible

**Figure 5:**
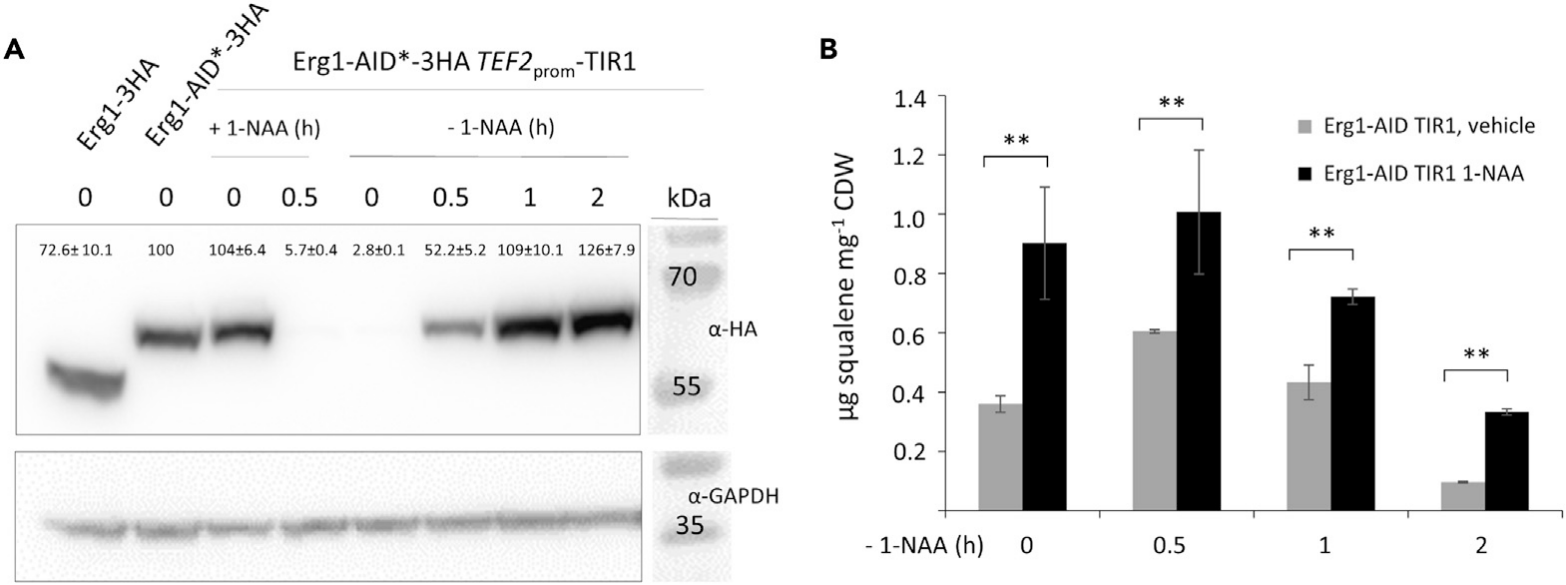
Depletion of Erg1 is readily reversible on the removal of 1-NAA (A) The CBS7435 strain expressing Erg1-AID∗-3HA and TIR1 from the *TEF2* promoter (yLL148) was grown to A_600_ ∼ 4 at 28°C, and degradation of Erg1-AID∗-3HA was induced by the addition of 0.5 mM 1-NAA for 30 min. Afterward, cells were washed twice with BMD medium, resuspended in BMD and samples taken at indicated time points prepared and analyzed by immunoblotting with anti-HA, as described under STAR Methods. As controls, CBS7435 strains expressing Erg1-3HA (yLL144) and Erg1-AID∗-3HA (yLL150) were used. GAPDH detected on the same immunoblots using anti-GAPDH antibody was used as a loading control. MW, marker proteins (kDa). Signal quantifications are presented as mean +/–SEM. (B) The same strain as in A was grown to A_600_ ∼ 4 at 28°C, and degradation of Erg1-AID∗-3HA was induced by the addition of 0.5 mM 1-NAA for 2 h. Afterward, cells were washed twice with BMD medium, resuspended in BMD, and samples taken at indicated time points were prepared and analyzed by GC-MS, as described under STAR Methods (three technical replicates were performed for each). Quantification of squalene was performed by comparing values to standard calibration curves, and all data were normalized by the internal standard cholesterol and cell dry weight (CDW). Squalene amounts ranging from 0 to 1.4 μg mg^−1^ CDW are represented on the y-axis. Error bars, SEM, ∗∗p value < 0.001, determined by two-tailed Student’s *t* test. *p* values were obtained for comparing 1-NAA treated samples with the respective vehicle controls for each time point.

One of the big advantages of the AID system is its reversibility (Nishimura et al., 2009). To determine the reversibility of AID-targeted degradation after removal of 1-NAA, we induced the degradation of Erg1-AID∗-3HA and then replaced the cultivation medium with fresh medium without 1-NAA. We found that expression of Erg1-AID∗-3HA was mostly recovered after 1 h (Figure 5A), confirming the quick reversibility of the system. The re-accumulation of Erg1-AID∗-3HA is immediately reflected in decreasing levels of squalene in the cell (Figure 5B).

## Discussion

**Figure 6:**
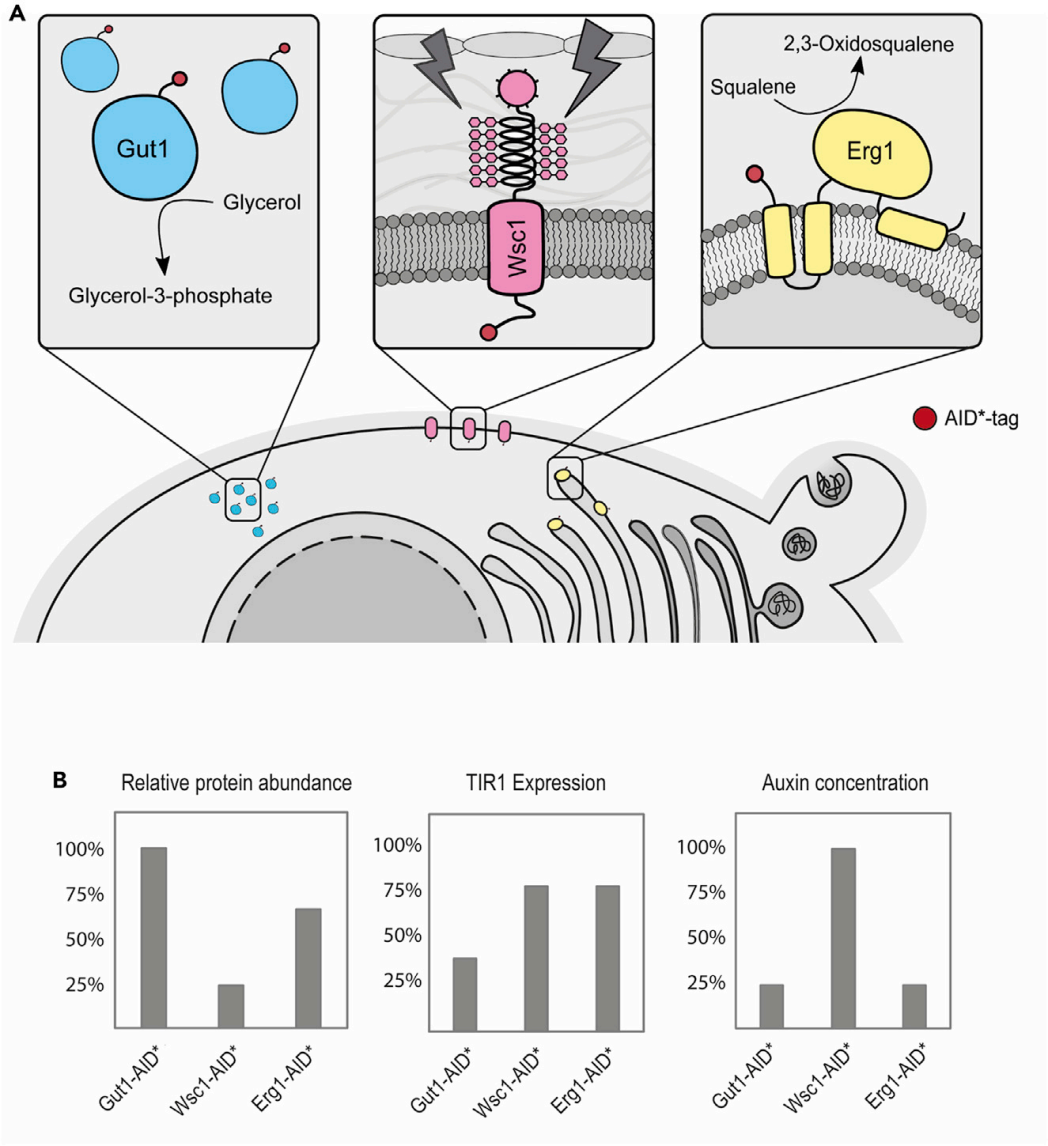
Degradation of target proteins depends on cellular localization (A) Overview of cellular localization of target proteins Gut1, Wsc1, and Erg1. (B) Simplified graphical illustration of relative protein abundance of Gut1-AID∗, Wsc1-AID∗, and Erg1-AID∗, and best conditions found for auxin-induced degradation of target proteins in respect to TIR1 expression levels and 1-NAA concentrations. The results presented in this illustration were concluded from Figure S4; Figures 2, 3, and 4; and Figure S1, respectively.

Here, we comprehensively characterized the usability of the auxin-inducible degron system in the yeast *K. phaffii*. Depending on the target protein, exposure of cells to 1-NAA for 10–30 min resulted in efficient protein depletion. With our approach, we show that varying promoters enable tunable expression of TIR1, which seems to have a target-specific effect on auxin-mediated degradation of AID∗-tagged proteins. We noticed that rather localization (Figure 6A) than abundance (Figure 6B) of the target protein plays a role in degradability. Even though Wsc1-AID∗-3HA and Erg1-AID∗-3HA are expressed at much lower levels than Gut1-AID∗-3HA (Figure S5), they require higher amounts of TIR1, and in case of Wsc1-AID∗-3HA, also higher auxin concentrations for efficient target degradation. As a soluble protein, TIR1 was shown to accumulate in the nucleus and cytoplasm and quickly relocates to the AID-tagged target protein once auxin is present in the cell (Holland et al., 2012). This probably favors the interaction of TIR1 with other soluble proteins such as Gut1. In contrast, membrane-associated proteins like Wsc1 and Erg1 are presumably less accessible to TIR1 degradation, most probably owing to spatial effects and reduced direct interaction.

Ohsawa et al. recently published a study focusing on the role of Wsc-type sensors of *K. phaffii* strain CBS7435 in sensing methanol in the medium (Ohsawa et al., 2017), but the study neither included a *wsc1*Δ *wsc2*Δ *wsc3*Δ triple knockout strain nor addressed the fact that no homologous genes of the *S. cerevisiae* Mid2-type cell wall sensors, *Sc*Mid2 and *Sc*Mtl1, can be found in the *K. phaffii* genome. As we did not succeed in additionally deleting *WSC1* in a *wsc2-3*Δ deletion strain, we established a complementation assay, where we show that a *wsc1*Δ *wsc2*Δ *wsc3*Δ triple knockout was unable to lose a highly unstable plasmid co-expressing *WSC1* even in the absence of selection pressure (Figure S3). Auxin-induced degradation of Wsc1 in a *wsc2*Δ *wsc3*Δ mutant strain resulted in severe growth defects when applying OsTIR1 and AID2, whereas Wsc1 was degraded rapidly and efficiently under all conditions tested (Figure 3). The biggest advantage of AID2 clearly is the reduced basal degradation of the target protein (Watson et al., 2021; Yesbolatova et al., 2020). Although basal degradation of Wsc1 was fully absent when applying AID2 (Figure 3D), the growth phenotype observed for auxin-induced degradation of Wsc1 in a *wsc2-3*Δ double delete strain was similar for AID (using 1-NAA and IAA) and AID2 (using 5-Ph-IAA). This either indicates that neither AID nor AID2 is able to fully eliminate each and every single molecule of Wsc1 and that the remaining pool is still sufficient to rescue cell growth, or that *K. phaffii* cells devoid of Wsc-type sensors are still able to exhibit slight cellular growth.

A more applied experiment we successfully applied the AID system for was the production of squalene, a pharmaceutically important intermediate product of the ergosterol biosynthetic pathway. This shows that the AID system could also find use in industrial applications. In the same experiment, we could confirm that the AID system is reversible: after the removal of 1-NAA, the recovery of the targeted protein starts immediately (Figure 5B). Literature gives different results about the velocity of reversibility, from immediate recovery up to several generations (sometimes up to 8 h (Camlin and Evans, 2019)). Overall, this seems to be strongly dependent on the protein detection method chosen (single molecule detection (Papagiannakis et al., 2017) versus bulk analysis by fluorescence or immunodetection (Farr et al., 2014; Holland et al., 2012)) as well as protein expression, abundance and stability (Papagiannakis et al., 2017) and the auxin type used (Camlin and Evans, 2019). In human cells, leaky degradation and re-expression of the target protein could get enhanced by the addition of 200 μM auxinole, an OsTIR1 inhibitor (Yesbolatova et al., 2020). For *K. phaffii* this does not seem to be necessary based on the quick recovery of protein re-expression, which we could (I) show by increasing Erg1 protein levels after removal of 1-NAA, and (II) by quickly decreasing squalene levels once Erg1 levels recover.

The AID technology is also a great tool to study essential genes. In *S. cerevisiae*, ∼19% of the genes were found to be essential, based on the fact that spores carrying a deletion allele of these genes failed to germinate and form colonies under standard laboratory conditions – a method known as tetrad analysis (Giaever et al., 2002). In contrast, there is very little knowledge about whether or not a gene is essential in *K. phaffii*, because tetrad analysis is extremely hard to implement as mating and sporulation are less effective and stable in this yeast (reviewed by (Bernauer et al., 2021)). A promising study analyzed the efficiency of random transposon insertion into the *K. phaffii* genome and, thereby made predictions about whether a gene is essential or not (Zhu et al., 2018). However, these predictions are very vague, often oddly differ from what has been published for *S. cerevisiae*, and need further experimental proof. The use of conditional mutants, e.g. generated by AID^∗^-tagging of a gene of interest can greatly facilitate the detection and functional analysis of essential proteins, particularly those of previously unknown function.

Our experiments prove that AID∗-tagged proteins can be degraded efficiently by applying weak to intermediate constitutive expression of OsTIR1 in *K. phaffii* from integrated plasmids, whereas basal degradation did not dramatically affect any of our chosen target proteins. However, when working with more sensitive targets, we recommend to apply AID2, which also efficiently eliminated basal degradation in our hands. Overall, the AID system is a simple and highly versatile tool, providing excellent means to dynamically perturb biological systems. By exploring the optimal experimental parameters for the AID system and by revealing its advantages and obstacles, our work adds value to a powerful tool for yeast researchers. The characterization, insights, and solutions provided in this work will allow yeast researchers to adapt this powerful tool to target any protein of interest in *K. phaffii* for immediate, fast, and reversible removal from the cell. This technique will enable researchers to tackle scientific questions on a whole new level and facilitate the achievement of many research goals not realizable with present techniques yet.

### Limitations of the study

Even though CRISPR/Cas has been established for targeted engineering of the *K. phaffii* genome, correct DNA insertions can still be tricky, especially when working with wild-type and not a *ku70*Δ knockout strain (Näätsaari et al., 2012). Hence, multiple clones had to be screened in order to obtain strains expressing AID-tagged proteins.

Degradation of the selected target proteins seemed efficient on all immunoblots; however, there could still be residual amounts of protein that could still compensate for the phenotype that would arise from the respective gene knockout (e.g. as potentially observed for Wsc1; or Gut1 and Erg1 when TIR1 was expressed from the *PGK1* promoter).

## STAR★Methods

### Key resources table

**Table:** 

REAGENT or RESOURCE	SOURCE	IDENTIFIER
**Antibodies**		

ANTI-FLAG® M2-Peroxidase (HRP) antibody	Sigma-Aldrich	Cat#A8592; RRID:AB_439702
Peroxidase-conjugated anti-HA 3F10 from rat	Roche	Cat# ROCHE 12 013 819 001; RRID:AB_390917
Anti-GAPDH	Institute of Biochemistry, Graz University of Technology, Austria	(Grillitsch et al., 2014)
goat Anti-Rabbit IgG–Peroxidase antibody	Sigma-Aldrich	Cat# A9169; RRID:AB_258434

**Chemicals, peptides, and recombinant proteins**		

1-NAA	Sigma-Aldrich	Cat# N0640
5-Ph-IAA	MedChemExpress	Cat# HY-134653
Terbinafine	Sigma Aldrich	Cat# T8826
NuPAGE™ sample buffer	Thermo Fisher Scientific Inc.	Cat# NP0007
PonceauS solution	Sigma-Aldrich	Cat# P7170
Clarity Max Western ECL Substrate	Bio-Rad	Cat# 1705062
PageRuler™ pre-stained protein ladder	Thermo Scientific™	Cat# 26616
Cholesterol	Sigma-Aldrich	Cat# C8667
N,O-bis (trimethylsilyl)trifluoroacetamide	Sigma-Aldrich	Cat# 15222
Pyridine	Sigma-Aldrich	Cat# 270970
Squalene	Sigma-Aldrich	Cat# S3626

**Experimental models: Organisms/strains**		

Strain: CBS7435a; Geno-type: *his4*Δ	Näätsaari et al. (2012)	https://pubmed.ncbi.nlm.nih.gov/22768112/
Strain: yLL132; Geno-type: CBS7435 *his4*Δ *PGK1*prom -TIR1-FLAG	This study	Pichia pool ID yLL132
Strain: yLL108; Geno-type: CBS7435 *his4*Δ *TEF2*prom -TIR1-FLAG	This study	Pichia pool ID yLL108
Strain: yLL109; Geno-type: CBS7435 *his4*Δ *HTA1*prom -TIR1-FLAG	This study	Pichia pool ID yLL109
Strain: yAEA398; Geno-type: CBS7435 *his4*Δ *GUT1*_prom_::*GUT1*-3HA	This study	Pichia pool ID yAEA398
Strain: yLL116; Geno-type: CBS7435 *his4*Δ *GUT1*_prom_::*GUT1*-AID∗-3HA	This study	Pichia pool ID yLL116
Strain: yLL118; Geno-type: CBS7435 *his4*Δ *GUT1*_prom_::*GUT1*-AID∗-3HA *PGK1*prom-TIR1	This study	Pichia pool ID yLL118
Strain: yLL120; Geno-type: CBS7435 *his4*Δ *GUT1*_prom_::*GUT1*-AID∗-3HA *TEF2*prom-TIR1	This study	Pichia pool ID yLL120
Strain: yLL122; Geno-type: CBS7435 *his4*Δ *GUT1*_prom_::*GUT1*-AID∗-3HA *HTA1*prom-TIR1	This study	Pichia pool ID yLL122
Strain: yLL104; Geno-type: CBS7435 *his4*Δ *wsc1*::Hyg *wsc2*::*HIS4*	This study	Pichia pool ID yLL104
Strain: yLL146; Geno-type: CBS7435 *his4*Δ *wsc2-3*::*HIS4 WSC1*_prom_::*WSC1*-3HA	This study	Pichia pool ID yLL146
Strain: yAEA400; Geno-type: CBS7435 *his4*Δ *wsc2-3*::*HIS4 WSC1*_prom_::*WSC1*-AID∗-3HA	This study	Pichia pool ID yAEA400
Strain: yLL141; Geno-type: CBS7435 *his4*Δ *wsc2-3*::*HIS4 WSC1*_prom_::*WSC1*-AID∗-3HA *PGK1*_prom_-TIR1-FLAG	This study	Pichia pool ID yLL141
Strain: yLL142; Geno-type: CBS7435 *his4*Δ *wsc2-3*::*HIS4 WSC1*_prom_::*WSC1*-AID∗-3HA *TEF2*_prom_-TIR1-FLAG	This study	Pichia pool ID yLL142
Strain: yLL143; Geno-type: CBS7435 *his4*Δ *wsc2-3*::*HIS4 WSC1*_prom_::*WSC1*-AID∗-3HA *HTA1*_prom_-TIR1-FLAG	This study	Pichia pool ID yLL143
Strain: yLB215; Geno-type: CBS7435 *his4*Δ *wsc2-3*::*HIS4 WSC1*_prom_::*WSC1*-AID∗-3HA *TEF2*_prom_-TIR1^F74G^-FLAG	This study	Pichia pool ID yLB215
Strain: yLB218; Geno-type: CBS7435 *his4*Δ *wsc2-3*::*HIS4 WSC1*_prom_::*WSC1*-AID∗-3HA *HTA1*_prom_-TIR1^F74G^-FLAG	This study	Pichia pool ID yLB218
Strain: yLL100; Geno-type: CBS7435 *his4*Δ *WSC1*_prom_::*WSC1*-3HA	This study	Pichia pool ID yLL110
Strain: yLL151; Geno-type: CBS7435 *his4*Δ *wsc2-3*::*HIS4 WSC1*_prom_::*WSC1*-AID∗-3HA *TEF2*_prom_-TIR1-FLAG *WSC1*_prom_-*WSC1*:: *his4*Δ	This study	Pichia pool ID yLL151
Strain: yLL120; Geno-type: CBS7435 *his4*Δ *wsc2-3*::*HIS4 WSC1*_prom_::*WSC1*-AID∗-3HA *TEF2*_prom_- TIR1-FLAG *WSC3*_prom_-*WSC3*:: *his4*Δ	This study	Pichia pool ID yLL120
Strain: yLL145; Geno-type: CBS7435 *his4*Δ *wsc2-3*::*HIS4 WSC1*_prom_::*WSC1*-AID∗-3HA *TEF2*_prom_- TIR1-FLAG *TEF2*_prom_-*RHO1*:: *his4*Δ	This study	Pichia pool ID yLL145
Strain: yLL144; Geno-type: CBS7435 *his4*Δ *ERG1*-3HA	This study	Pichia pool ID yLL144
Strain: yLL150; Geno-type: CBS7435 *his4*Δ *ERG1*-AID∗-3HA	This study	Pichia pool ID yLL150
Strain: yLL147; Geno-type: CBS7435 *his4*Δ *ERG1*-AID∗-3HA *PGK1*_prom_-TIR1-FLAG	This study	Pichia pool ID yLL147
Strain: yLL148; Geno-type: CBS7435 *his4*Δ *ERG1*-AID∗-3HA *TEF2*_prom_-TIR1-FLAG	This study	Pichia pool ID yLL148

**Oligonucleotides**		

Fw(sg_*GUT1*tt) ACGAAACGAGTAAGC TCGTCATGGAAATCGATGGATATCG GTTTTAGAGCTAGAAATAGC	This study	N/A
Rv(sg_GUT1tt) GAGCTTACTCGTTTCGTCCTCACGGAC TCATCAGATGGAATTTGATTTGTTTAGGTAACT	This study	N/A
Rv(pPpCas9_rest) GGGCATCACAATCATGGAGC	This study	N/A
Fw(pPpCas9_rest) CCTCGAGAAAGTCGATGGGG	This study	N/A
Fw(TIR1F74G) GACTGTTAAAGGTAAACCTCATGGTGCTGATT TCAATTTGGTTCCACC	This study	N/A
Rv(TIR1F74G) GGTGGAACCAAATTGAAATCAGCACCATGA GGTTTACCTTTAACAGTC	This study	N/A

**Recombinant DNA**		

pPpT4- *PGK1*_prom_-TIR1-FLAG	This study	Addgene ID 189724
pPpT4- *TEF2*_prom_-TIR1-FLAG	This study	Addgene ID 189725
pPpT4- *HTA1*_prom_-TIR1-FLAG	This study	Addgene ID 189726
pPpT4- *TEF2*_prom_-TIR1^F74G^-FLAG	This study	Addgene ID 189727
pPpT4- *HTA1*_prom_-TIR1^F74G^ -FLAG	This study	Addgene ID 189728

### Resource availability

#### Lead contact

Further information and requests for resources and reagents should be directed to and will be fulfilled by the lead contact, Anita Emmerstorfer-Augustin (emmerstorfer-augustin@tugraz.at).

#### Materials availability

All strains and plasmids described in this study can be obtained from the Pichia Pool strain collection at the Institute of Molecular Biotechnology at Graz University of Technology (Graz, Austria). All plasmids expressing TIR1 were additionally deposited to Addgene (pAEA454, pLL002, pLL001, pLB219 and pLB220). No new unique reagents were generated in this study.

### Experimental model and subject details

#### K. phaffii

In this study, the *K. phaffii* strain CBS7435 *his4*Δ (NRRL Y-11430, ATCC 76273) (Näätsaari et al., 2012) was used as wild-type strain for further engineering.

All yeast strains used in this study (key resources table) were either grown on buffered minimal dextrose (BMD) media [2% glucose, 13.4 g L^−1^ Yeast Nitrogen Base (without amino acids), 4∗10^−5^% biotin, 0.4% histidine to permit growth of auxotrophs, and 100 mM phosphate buffer pH 6], or on buffered minimal glycerol (BMG) media [1% glycerol, 13.4 g L^−1^ Yeast Nitrogen Base (without amino acids), 4∗10^−5^% biotin, 0.4% histidine to permit growth of auxotrophs, and 100 mM phosphate buffer pH 6] (Invitrogen, 2014). Cultures were propagated at 28 °C, unless indicated otherwise. For preparation of glycerol stocks, 5 mL of YPD [1% yeast extract, 2% peptone, 2% glucose] were inoculated with cells, incubated in a shaker at 28 °C overnight, pelleted at 5,000 rpm for 5 min, resuspend in 1 mL of YPD and 500 μL of 50% glycerol and frozen at −80°C.

### Method details

#### Cloning of pPpKC2 repair plasmids and pPpHyg-Cas9 targeting plasmids

As basis for all repair plasmids, we used the vector pPpKC2 (Ahmad et al., 2019). The KanMX marker has been removed by cutting the vector with *BamH*I and *Kpn*I and instead, repair cassettes were cloned into the linearized vector. Repair cassettes were designed the following way: 500–1000 bp long up- and downstream-regions of the CRISPR/Cas9 gene-specific targeting sites flank in-frame either an AID∗-3HA- or an 3HA-tag (amplified from pHyg-AID∗-6HA (Morawska and Ulrich, 2013)) followed by a TAA stop codon and an *ARG4* transcription termination sequence for efficient, marker-less homologous recombination of the repair cassette (Figure 1C). In case the sequence of the repair cassette still harbored the CRISPR/Cas9 targeting and PAM sequence, the PAM sequence was mutated to a silent mutation in order to avoid renewed Cas9-inflicted double-strand breaks of the repaired locus. PCR amplification was performed using Phusion™ DNA polymerase (Thermo Fisher Scientific Inc., St. Leon-Rot, Germany), plasmids were cloned applying Gibson assembly (Gibson et al., 2009) and all constructs were verified by restriction analysis and DNA sequencing. Repair cassettes were cut out from readily cloned vectors using *Smi*I and gel purified.

#### Construction of strains expressing HA- and AID∗-HA tagged target genes applying CRISPR/Cas9

pPpHyg-Cas9 plasmids were derived from pPpT4-pHTX-PARS1-*Hs*Cas9 (Weninger et al., 2018). The original Zeocin resistance gene in pPpT4-pHTX-PARS1-*Hs*Cas9 has been replaced by a Hygromycin resistance gene by cutting the plasmid and the resistance gene with restriction enzymes *Mfe*I and *Nco*I. In this vector, sgRNA targeting sequences were customized to target specific genes (Table S2) applying a simple Gibson assembly approach. Overlapping, 60 bp long forwards and reverse primers were designed harboring exchanged 20 bp for sgRNA binding and the 6 reverse complementary bp of the Hammerhead sequence. To give an example, in case of *GUT1* (sgRNA binding site and PAM sequence listed in Table S2), the Fw(sg_*GUT1*tt) was designed as forward primer and Rv(sg_GUT1tt) as reverse primer. Two other short primers were additionally generated to bind the vector backbone of pPpHyg-Cas9, namely Rv(pPpCas9_rest) and Fw(pPpCas9_rest) (full primer sequences are provided in the key resources table). By using pPpHyg-Cas9 as template, two PCR fragments were generated applying Fw(sg_*GUT1*tt) and Rv(pPpCas9_rest), and Fw(pPpCas9_rest) and Rw(sg_*GUT*1tt), the two PCR products gel purified and assembled using Gibson Assembly. The same strategy has been used to target *WSC1* and *ERG1*, whereas only primers Fw(sg_*GUT*1tt) and Rv(sg_*GUT1*tt) had to be adapted. A summary of the sgRNA binding site chosen for each target is provided (Table S2).

The pPpZeo-Cas9 plasmid targeting *WSC3* for frameshift mutation was derived from pPpT4-pHTX-PARS1-*Hs*Cas9 (Weninger et al., 2018). For the exchange of the sgRNA targeting sequence, the same strategy as for targeting *GUT1*tt was used. The targeting sequence of *WSC3* is shown in Table S2. In order to achieve complementation of *wsc1*Δ, the promoter and coding region of *WSC1* and an *ARG4* transcription termination sequence were assembled into the *Smi*I site of the pPpZeo-Cas9 plasmid targeting *WSC3* using Gibson assembly.

For gene editing of *GUT1*, *WSC1* and *ERG1*, CRISPR/Cas9 was used following the protocol of Weninger et al. with slight modifications (Weninger et al., 2016). Briefly, cells were grown overnight in YPD at 28°C, diluted to an OD_600_ of 0.1 and cultivated at 28°C until they reached an OD_600_ of 0.7–1. Cells were then prepared for electrotransformation as described by Cereghino et al. (Lin-Cereghino et al., 2005). For CRISPR/Cas genome editing, 100 ng of pPpHyg-Cas9 plasmid and 500 ng of the respective repair cassette were co-tranformed into cells. Correct integration of cassettes into the yeast genome was verified by cPCR and sequencing.

#### Cloning of pPpT4-TIR1 expression plasmids and strain construction

First, the vector backbone of pPpT4 (Näätsaari et al., 2012) was amplified by PCR. Second, promoter sequences of *PGK1* (protein ID CCA37205.1), *TEF2* (homologue of *S. cerevisiae TEF1*) (protein ID CCA37646.1) and *HTA1* (Protein ID SCV12077.1) were amplified from the *K. phaffii* genome. And third, TIR1 was amplified from plasmid pMK200 containing yeast codon optimized TIR1 (NBRP ID: BYP7569; Masato Kanemaki) and an FLAG-tag was added C-terminally in order to monitor expression of TIR1. The three PCR products were assembled using Gibson assembly. For cloning of the TIR1^F74G^ variant, an optimized QuikChange mutagenesis protocol was applied (Edelheit et al., 2009) using primers Fw(TIR1F74G) and Rv(TIR1F74G) (full primer sequences are provided in the key resources table) with pLL002 as template. All constructs were verified by restriction analysis and DNA sequencing and the plasmid maps (Table S1) can be downloaded as Data S1.

For ectopic expression of TIR1 and TIR1^F74G^, plasmids pPpT4-*PGK1*_prom_-TIR1-FLAG, pPpT4-*TEF2*_prom_-TIR1^F74G^-FLAG, and pPpT4-*HTA1*_prom_-TIR1-FLAG were digested with *Smi*I, purified and transformed into *K. phaffii* cells for integration into the 3′ UTR of the *ARG4* locus following the condensed protocol by Lin-Cereghino (Lin-Cereghino et al., 2005). In order to avoid multiple, random integrations of the TIR1-expression cassettes into the *K. phaffii* genome, we used 50–100 ng of purified cassette DNA for transformation.

#### *WSC1* complementation assays

For testing viability of a *wsc1*Δ *wsc2*Δ *wsc3*Δ triple knockout strain, a wild type strain and an otherwise isogenic derivative carrying deletions of *WSC1* and *WSC2* were transformed with plasmids pPpCas9-Zeo-*gut1*Δ (Weninger et al., 2016) and pPpCas9-Zeo-*wsc3*Δ_*WSC1* (pAEA465) and transformants were selected on YPD-Zeo plates. After 3 days of incubation at 28°C, single colonies were transferred to fresh YPD plates by streak plate method and incubated for 24 h at 28°C. Single colonies form these plates were transferred again to YPD and YPD-Zeo plates and streaked in small patches. Correct targeting and frameshift-mutation of the *WSC3* locus was confirmed by cPCR and sequencing of the PCR product.

#### Incubation with auxin or terbinafine

Auxin-induced degradation of AID∗-tagged proteins was initiated in TIR1-expressing cells growing in BMD or BMG buffered with 100 mM K_2_HPO_4_/KH_2_PO_4_ (pH 6) at an A_600_ of ∼4, by addition of 1-NAA (Sigma-Aldrich) (0.5 mM final concentration), a cell-permeable synthetic auxin (Robert et al., 2010). Alternatively, 5-Ph-IAA (MedChemExpress) was used at a concentration of 5 or 20 μM (Yesbolatova et al., 2020). For inhibition with terbinafine, cultures were grown at 28°C in BMD or BMG buffered with 100 mM K_2_HPO_4_/KH_2_PO_4_ (pH 6) to A_600_ of ∼4, and terbinafine (Sigma-Aldrich) (0.1 μg mL^−1^ final concentration) was added. For reversibility assays, cells were incubated for 30 min (in case of immunoblot samples), or 2 h (in case of squalene analysis) with 1-NAA were washed with BMG medium twice, resuspended in BMG medium and incubated at 28°C for the times indicated.

For spot assays to assess cell growth and viability, cell cultures were pregrown overnight to saturation in BMD buffered with 100 mM K_2_HPO_4_/KH_2_PO_4_ (pH 6) and diluted to A_600nm_ = 1 in a 96-well microtiter plate, and then those wells were subjected to serial 5-fold dilutions. Samples of each dilution were spotted, using a Steers-type multipronged inoculator, onto agar plates containing phosphate-buffered BMD or BMG medium and containing either 0.25–1 mM 1-NAA in DMSO, 5 μM 5-Ph-IAA in DMSO, or an equivalent volume of DMSO alone (control plates). Sorbitol was added to a final concentration of 1 M, and an equivalent volume of DMSO was used in control plates. Plates were incubated at 28°C and typically photographed after 72 h.

#### Immunoblot analysis

Cells from middle exponential-phase cultures (4 A_600nm_ equivalents) were collected by brief centrifugation, lysed in 300 μL 1.85 M NaOH containing 7.4% β-mercaptoethanol, and proteins in the resulting lysate were precipitated by addition of 300 μL 50% trichloroacetic acid on ice for 60 min. Precipitated proteins were collected by centrifugation for 5 min in the microfuge at 4 °C and maximum rpm and washed with ice-cold water to remove excess trichloroacetic acid. The precipitated proteins were solubilized in 50 μL of 1× NuPAGE™ sample buffer (Thermo Fisher Scientific Inc., St. Leon-Rot, Germany) supplemented with 4% β-mercaptoethanol and 30% 1 M Tris. The resulting solubilized protein was heated at 65°C for 10 min, except for lysates containing Wsc1-HA and Wsc1-AID∗-HA, samples briefly centrifuged at 10,000 rpm for 20 s, and 12 μL of the supernatant resolved by NuPAGE™ Mini Protein Gels (12%, Bis-Tris, 1.0 mm; Thermo Fisher Scientific Inc., St. Leon-Rot, Germany) at 160 V. Resolved proteins were transferred electrophoretically to a nitrocellulose membrane using a wet transfer apparatus (NuPAGE™, Thermo Fisher Scientific Inc., St. Leon-Rot, Germany) (Towbin et al., 1979) and efficient blotting was checked using PonceauS (Sigma-Aldrich) (Figure S5). For removal of PonceauS, protein blots were washed with ddH_2_O until the staining disappeared and incubated for 1 h in TBST-milk (2.5%). After blocking, the membranes were incubated overnight at 4°C in the same blocking buffer with an appropriate primary antibody (at the indicated dilution): mouse ANTI-FLAG® M2-Peroxidase (HRP) antibody (1:4000; Sigma Aldrich); peroxidase-conjugated anti-HA 3F10 from rat (1:2500; Roche) and rabbit anti-GAPDH (1:5000; Institute of Biochemistry, Graz University of Technology, Austria) (Grillitsch et al., 2014). After washing with TBST (three times with ≥10 mL), membranes were either used directly for immunodetection, or incubated with an appropriate HRP-conjugated secondary antibody - goat Anti-Rabbit IgG–Peroxidase antibody A9169 (1:10,000, Sigma Aldrich, Vienna, Austria), and then washed with TBST (three times with ≥10 mL). Enhanced chemiluminescent signal detection (Clarity Max Western ECL Substrate, Bio-Rad) was used to visualize immunoreactive bands. Molecular weight marker used was the PageRuler™ pre-stained protein ladder (Thermo Scientific™). Quantifications of immunoblot signal intensities were done using Fiji (Schindelin et al., 2012).

#### Squalene analysis

For quantification of squalene, cell pellets corresponding to 15 OD_600_ units were essentially prepared for gas chromatography analysis as described before (Emmerstorfer-Augustin et al., 2019). First, cell pellets were resuspended in 600 μL of methanol, 400 μL of 0.5% pyrogallol in methanol, and 400 μL of 60% KOH, as well as 5 μL of a 2 mg mL^−1^ cholesterol (Sigma-Aldrich, St. Louis, MO) solution in ethanol (internal standard). Next, samples were incubated for 2 h at 90°C in a sand bath and saponified lipids extracted two times with 1 mL of n-heptane. Dried extracts were dissolved in 10 μL of pyridine and subsequently derivatized with 50 μL of N,O-bis (trimethylsilyl)trifluoroacetamide (Sigma-Aldrich, St. Louis, MO). Samples were diluted with 200 μL of ethyl acetate and analyzed by gas chromatography-mass spectrometry (GC-MS) as described previously (Ott et al., 2005). Quantification of squalene was performed by comparing values to standard calibration curves, and all data were normalized by and cell dry weight (CDW) and the internal standard cholesterol.

### Quantification and statistical analysis

All results reported reflect – except where indicated otherwise – findings reproducibly made in at least three independent trials of each experiment shown. For determination of mean values and SEM, Excel was used. Student’s T-test implemented in Scipy, the scientific library for Python (https://scipy.org, SciPy v1.5.4), was used for calculations of p values (highlighted with two asterisks in the respective graphs) to test the hypothesis that groups have identical means. Sample sizes, number of biological and technical replicates performed (n), statistical analysis used, and if and how the values presented were normalized, are all described in the relevant figure legends.

## Data Availability

• All data reported in this paper (immunoblots, images of growth assays and GC-MS measurements) will be shared by the lead contact upon request. • This paper does not report original code. • Any additional information required to reanalyze the data reported in this paper is available from the lead contact upon request.

## Supplemental information

Document S1. Figures S1–S5 and Tables S1 and S2

Data S1. Vector files of all plasmids cloned in this study, related to STAR Methods

## Footnotes

Supplemental information can be found online at https://doi.org/10.1016/j.isci.2022.104888.
