# Supplementary material for: Applying the auxin-based degron system for the inducible, reversible and complete protein degradation in Komagataella phaffii
Source: iScience. 2022 Aug 6;25(9):104888. doi: 10.1016/j.isci.2022.104888 (PMC9420516; doi:10.1016/j.isci.2022.104888)
Supplement: Document S1. Figures S1–S5 and Tables S1 and S2 [file mmc1.pdf]

iScience, Volume *25*

Supplemental information

Applying the auxin-based degron system for the inducible, reversible and complete protein degradation in *Komagataella phaffii*

Leonie Lehmayer, Lukas Bernauer, and Anita Emmerstorfer-Augustin

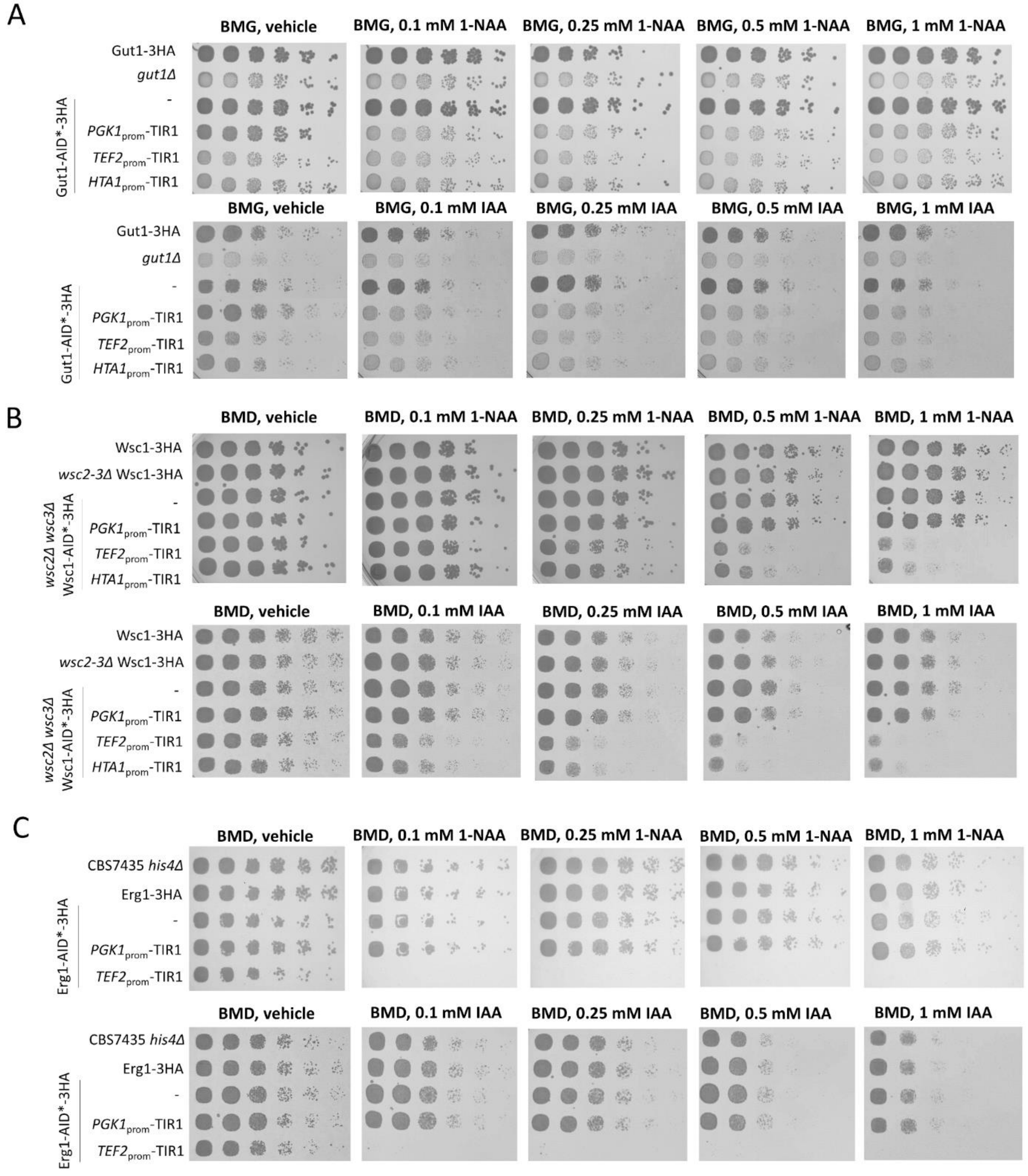

Supplementary Figure S1. Determination of ideal auxin concentrations for degradation of Gut1-AID*-3HA, Wsc1-AID*-3HA and Erg1-AID*-3HA, Related to Figure 2, Figure 3 and Figure 4. A) Strain yP322 (CBS7435 *gut1*Δ), a CBS7435 strain expressing either Gut1-3HA (yAEA398), Gut1-AID*-3HA (yLL116) or otherwise isogenic derivatives co-expressing TIR1 from *PGK1*$_{prom}$ (yLL118), *TEF2*$_{prom}$

(yLL120), or *HTA1*$_{prom}$ (yLL122) were cultivated as described in Materials and Methods, and then samples of a set of fivefold serial dilutions were spotted using a multiprong inoculator on an agar plate containing BMG with vehicle (DMSO) or different concentrations (0.1 mM, 0.25 mM, 0.5 mM and 1 mM) of 1-NAA or IAA, and, after incubation for 72 h at 28°C, the resulting growth was recorded. B.) A CBS7435 *wsc2*Δ *wsc3*Δ (*wsc2*-*3*Δ) knockout strain expressing either Wsc1-3HA (yLL146), Wsc1-AID*-3HA (yAEA400) or otherwise isogenic derivatives co-expressing TIR1 from *PGK1*$_{prom}$ (yLL141), *TEF2*$_{prom}$ (yLL142), or *HTA1*$_{prom}$ (yLL143) were prepared and spotted on BMD and BMD containing different concentrations of 1-NAA or IAA as explained under A. C.) A CBS7435 control strain, and CBS7435 strains expressing Erg1-3HA (yLL144), Erg1-AID*-3HA (yLL150), Erg1-AID*-3HA *PGK1*$_{prom}$-TIR1 (yLL147) and Erg1-AID*-3HA *TEF2*$_{prom}$-TIR1 (yLL148) were prepared and spotted on BMD with vehicle (DMSO) or different concentrations (0.1 mM, 0.25 mM, 0.5 mM and 1 mM) of 1-NAA or IAA, as explained under A.

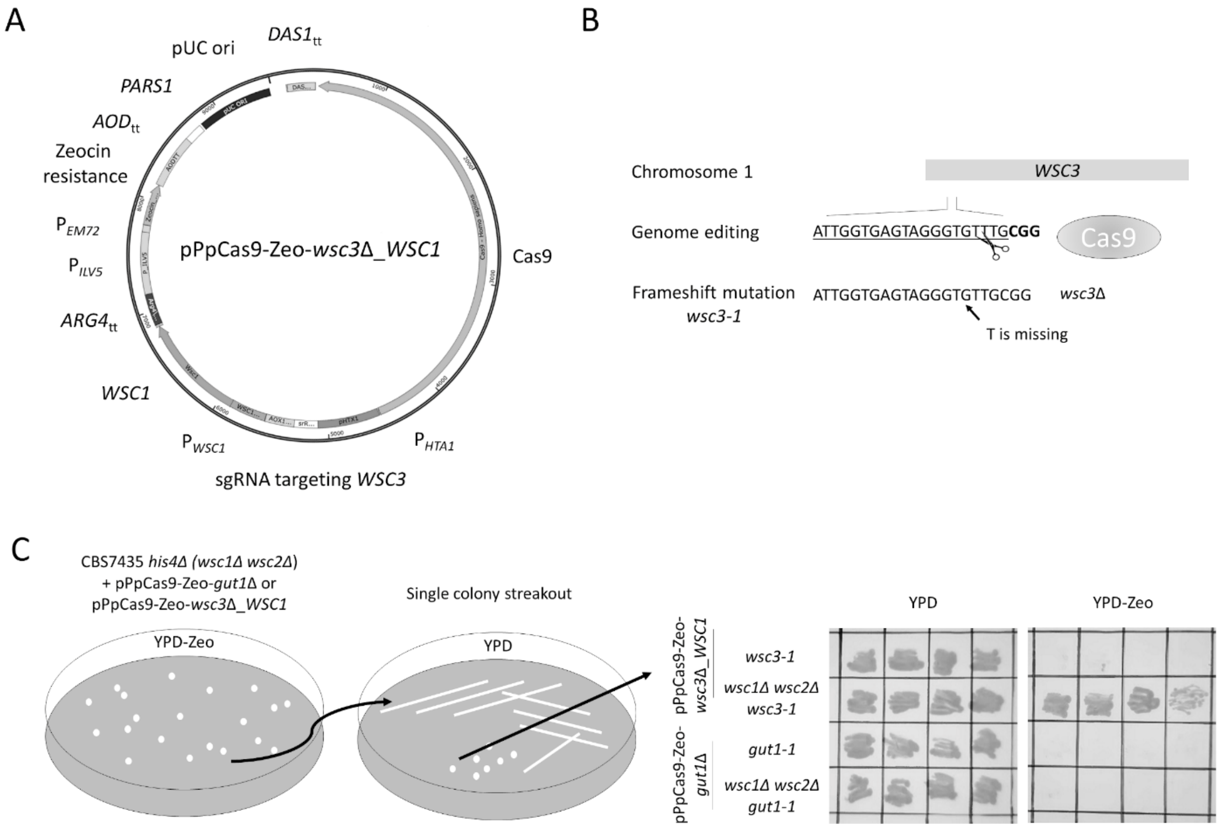

Supplementary Figure S2. A *wsc1*Δ *wsc2*Δ *wsc3*Δ triple knockout strain is most likely not viable, Related to Figure 3. A) In order to design a complementation experiment, a plasmid which generates a frameshift mutation in *WSC3* and simultaneously expresses one copy of *WSC1* from its endogenous promoter was designed. B) The frameshift mutation in *WSC3* was generated using the same CRISPR/Cas9 approach as described for HA- and AID*-tagging of *GUT1* (Supplementary information and Figure 1C). C) A wild type strain and an otherwise isogenic derivative carrying deletions of *WSC1* and *WSC2* were transformed with plasmids pPpCas9-Zeo-*gut1*Δ (Weninger et al., 2016) and pPpCas9-Zeo-*wsc3*Δ_*WSC1* (pAEA465) and transformants were selected on YPD-Zeo plates. After 3 days of incubation, single colonies were transferred to fresh YPD plates by streak plate method. Single colonies form these plates were transferred again to YPD and YPD-Zeo plates and streaked in small patches.

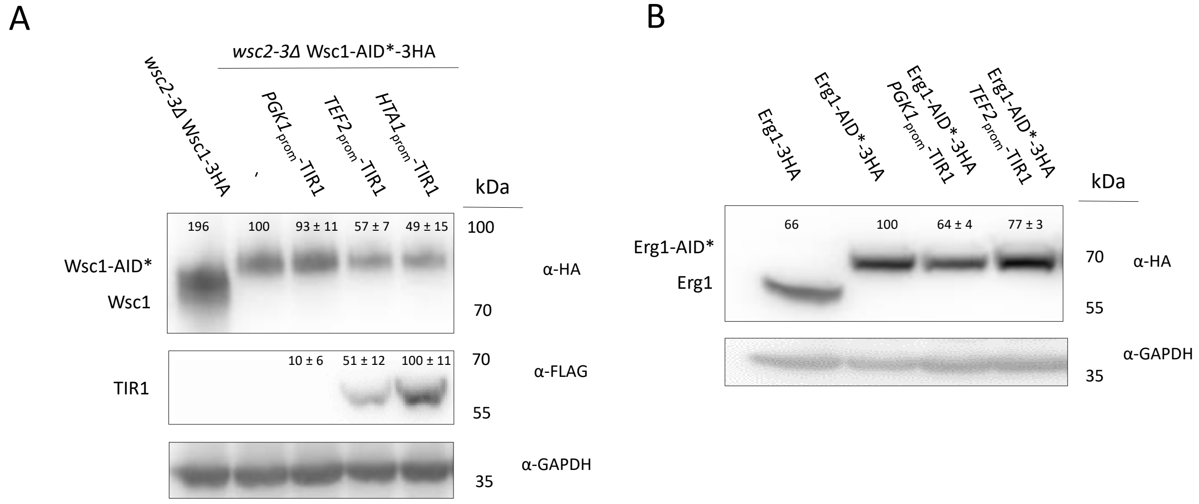

Supplementary Figure S3. Basal degradation of Wsc1-AID*-3HA and Erg1-AID*-3HA, Related to Figure 3 and Figure 4. A.) A CBS7435 *wsc2*Δ *wsc3*Δ (*wsc2-3*Δ) knockout strain expressing either Wsc1-3HA (yLL146), Wsc1-AID*-3HA (yAEA400) or otherwise isogenic derivatives co-expressing TIR1 from *PGK1*$_{prom}$ (yLL141), *TEF2*$_{prom}$ (yLL142), or *HTA1*$_{prom}$ (yLL143) were grown, prepared and analyzed by immunoblotting with anti-HA and anti-FLAG, as described under Materials and Methods. B.) CBS7435 strains expressing Erg1-3HA (yLL144), Erg1-AID*-3HA (yLL150), Erg1-AID*-3HA *PGK1*$_{prom}$-TIR1 (yLL147) and Erg1-AID*-3HA *TEF2*$_{prom}$-TIR1 (yLL148) were grown, prepared and analyzed by immunoblotting with anti-HA, as described under Materials and Methods. GAPDH detected on the same immunoblots using anti-GAPDH antibody was used as loading control. MW, marker proteins (kDa). Values above the lanes represent the percentage of relative protein levels with and without co-expression of TIR1 (average of three independent experiments with SEM).

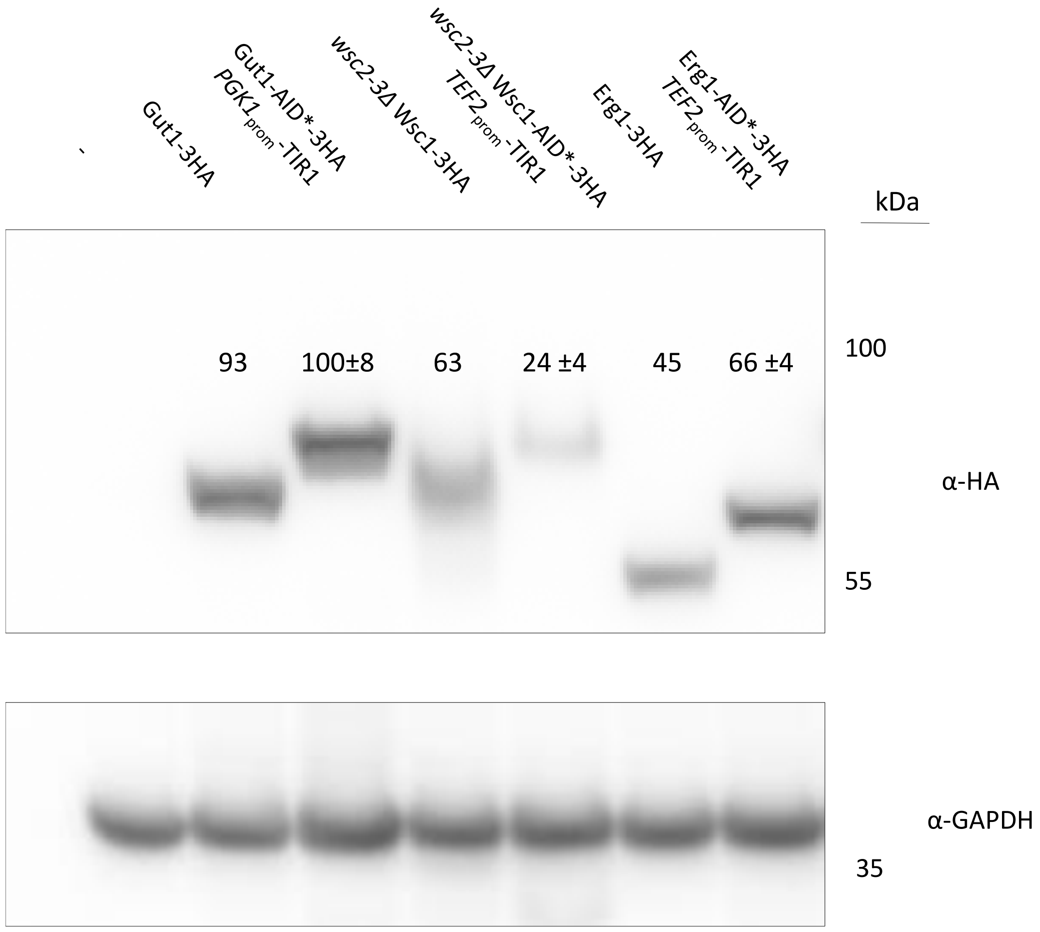

Supplementary Figure S4. Relative expression levels of Gut1-AID*-3HA, Wsc1-AID*-3HA and Erg1-AID*-3HA, Related to Figure 2, Figure 3 and Figure 4. A CBS7435 wild-type strain, CBS7435 *GUT1*-3HA (yAEA398), CBS7435 *GUT1*-AID*-3HA *PGK1*$_{prom}$-TIR1 (yLL118), CBS7435 *wsc2*Δ *wsc3*Δ (*wsc2-3*Δ) *WSC1*-3HA (yLL146), *WSC1*-AID*-3HA (yAEA400) or otherwise isogenic derivatives co-expressing TIR1 from *PGK1*$_{prom}$ (yLL141), *TEF2*$_{prom}$ (yLL142), or *HTA1*$_{prom}$ (yLL143) grown at 28°C were harvested at middle exponential phase, lysed, and proteins were extracted, resolved by SDS–PAGE, and analyzed by immunoblotting with anti-HA, as described under Materials and Methods. Loading control, GAPDH detected on the same immunoblots using anti-GAPDH antibody. MW, marker proteins (kDa). Values above the lanes represent the percentage of relative protein levels (average of three independent experiments with SEM).

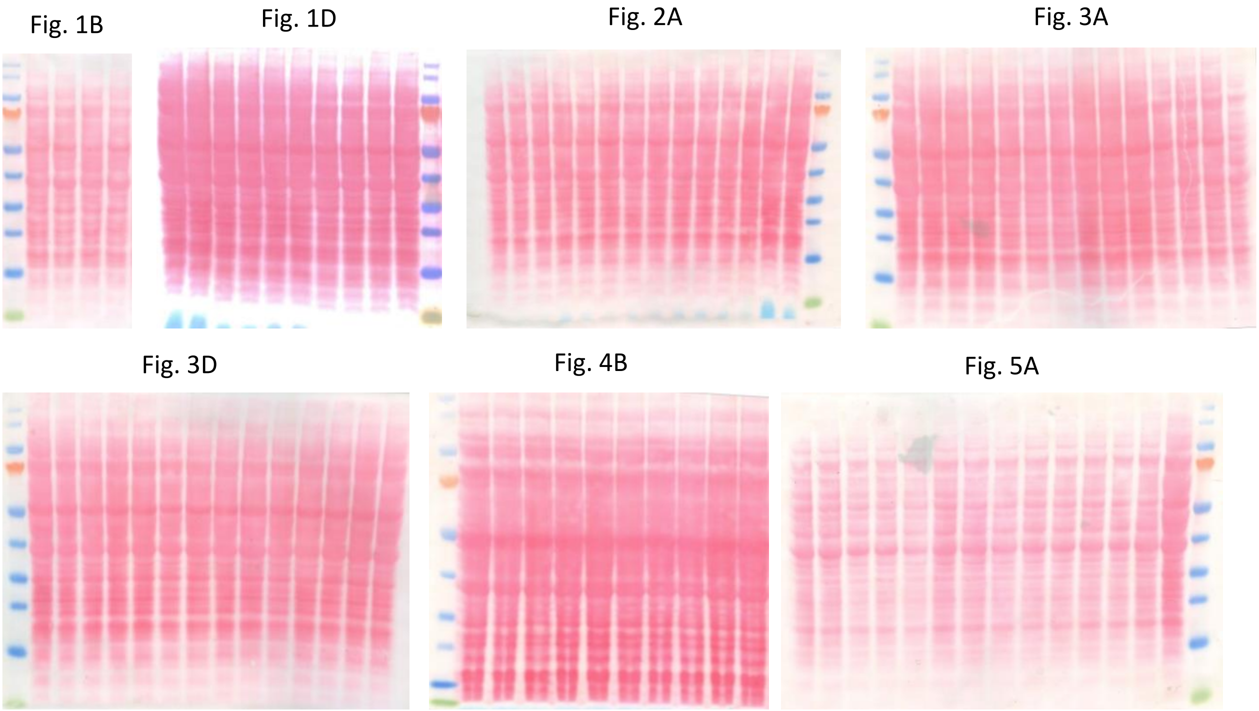

Supplementary Figure S5. PonceauS staining of immunoblots, Related to Figure 1B, Figure 1D, Figure 2A, Figure 3A, Figure 3D, Figure 4B, and Figure 5A. PonceauS stains all protein transferred from the gel to the membrane and proofs whether transfer worked efficiently. Molecular weight (MW) marker PageRuler™ Prestained Protein Ladder, 10 to 180 kDa (Thermo Fisher Scientific).

Table S1: Plasmids[a] used in this study, Related to STAR Methods.

Plasmid	Description	Reference or source
pAEA454	pPpT4- *PGK1*$_{prom}$-TIR1-FLAG	This study
pLL002	pPpT4- *TEF2*$_{prom}$-TIR1-FLAG	This study
pLL001	pPpT4- *HTA1*$_{prom}$-TIR1-FLAG	This study
pLB219	pPpT4- *TEF2*$_{prom}$-TIR1^{F74G}-FLAG	This study
pLB220	pPpT4- *HTA1*$_{prom}$-TIR1^{F74G} -FLAG	This study
pHyg-AID*-6HA	-	(Morawska and Ulrich, 2013)
pAEA452	pPpHyg-Cas9-*GUT1*tt	This study
pAEA453	pPpKC2-*GUT1*-3HA	This study
pLL003	pPpKC2-*GUT1*-AID*-3HA	This study
pAEA441	pPpHyg-Cas9-Wsc1tt	This study
pAEA451	pPpKC2-Wsc1-AID*-3HA	This study
pLL015	pPpKC2-his4-*WSC1*$_{prom}$-*WSC1*	This study
pAEA461	pPpKC2-his4-*WSC3*$_{prom}$-*WSC3*	This study
pLL005	pPpKC2-his4-*TEF2*$_{prom}$-*RHO1*	This study
pPpCas9-Zeo-*gut1*Δ	pPpT4-pHTX-*PARS1*-*Hs*Cas9-*gut1*	(Weninger et al., 2018)
pAEA465	pPpZeo-Cas9-*wsc3_WSC1*$_{prom}$-*WSC1*	This study
pLL011	pPpHyg-Cas9-*ERG1*tt	This study
pLL007	pPpKC2-*ERG1*-3HA	This study
pLL009	pPpKC2-*ERG1*-AID*-3HA	This study

[a] All plasmid maps are provided in the Supplementary Material.

Table S2: CRISPR/Cas9 targeting loci and sequences used to generate tagged or knockout strains, Related to STAR Methods.

	sgRNA binding site and PAM sequence	Generation of repair cassette
**Tagging or *GUT1***		
GUT1-3HA-repair cassette	ATGGAAATCGATGGATATCGCGG	Cut pAEA453 with *Smi*I and purify 3567 bp fragment
GUT1-AID*-3HA repair cassette	ATGGAAATCGATGGATATCGCGG	Cut pLL003 with *Smi*I and purify 3807 bp fragment
**Tagging of *WSC1***		
WSC1-3HA-repair cassette	TGAAGCAGATTACTCCAGGAAGG	Use pHyg-AID*-6HA as template for PCR with overlapping primers
WSC1-AID*-3HA repair cassette	TGAAGCAGATTACTCCAGGAAGG	Cut pAEA451 with *Smi*I and purify 1447 bp fragment
***WSC3* knockout**		
Frameshift mutation generated using pAEA465	ATTGGTGAGTAGGGTGTTTGCGG	-
**Expression of *WSC1* from *his4*Δ locus**		
WSC1$_{prom}$-*WSC1* integration cassette	TTATCAGTGAGTCAGTCATCAGG	Cut pLL015 with *Smi*I and purify 2637 bp fragment
**Expression of *WSC3* from *his4*Δ locus**		
WSC3$_{prom}$-*WSC3* integration cassette	TTATCAGTGAGTCAGTCATCAGG	Cut pAEA462 with *Smi*I and purify 3518 bp fragment
**Expression of *RHO1* from *his4*Δ locus**		
TEF2$_{prom}$-*RHO1* integration cassette	TTATCAGTGAGTCAGTCATCAGG	Cut pLL005 with *Smi*I and purify 2269 bp fragment
**Tagging or *ERG1***		
3HA-repair cassette	TCCCTATTTATGGGACGAACTGG	Cut pLL007 with *Smi*I and purify 2313 bp fragment
AID*-3HA repair cassette	TCCCTATTTATGGGACGAACTGG	Cut pLB009with *Smi*I and purify 2553 bp fragment
